# Constraints on Optimising Encoder-Only Transformers for Modelling Sign Language with Human Pose Estimation Keypoint Data

**DOI:** 10.3390/jimaging9110238

**Published:** 2023-11-02

**Authors:** Luke T. Woods, Zeeshan A. Rana

**Affiliations:** 1 Digital Aviation Research and Technology Centre (DARTeC), Cranfield University, Cranfield, Bedfordshire MK43 0AL, UK; 2 Leidos Industrial Engineers Limited, Unit 3, Bedford Link Logistics Park, Bell Farm Way, Kempston, Bedfordshire MK43 9SS, UK; 3 Centre for Aeronautics, School of Aerospace, Transport and Manufacturing (SATM), Cranfield University, Cranfield, Bedfordshire MK43 0AL, UK; zeeshan.rana@cranfield.ac.uk

**Keywords:** sign language recognition, human pose estimation, classification, computer vision, deep learning, machine learning, supervised learning, regularisation, data augmentation, 68T10, 68T45, 68T50

## Abstract

Supervised deep learning models can be optimised by applying regularisation techniques to reduce overfitting, which can prove difficult when fine tuning the associated hyperparameters. Not all hyperparameters are equal, and understanding the effect each hyperparameter and regularisation technique has on the performance of a given model is of paramount importance in research. We present the first comprehensive, large-scale ablation study for an encoder-only transformer to model sign language using the improved Word-level American Sign Language dataset (WLASL-alt) and human pose estimation keypoint data, with a view to put constraints on the potential to optimise the task. We measure the impact a range of model parameter regularisation and data augmentation techniques have on sign classification accuracy. We demonstrate that within the quoted uncertainties, other than $\ell_{2}$ parameter regularisation, none of the regularisation techniques we employ have an appreciable positive impact on performance, which we find to be in contradiction to results reported by other similar, albeit smaller scale, studies. We also demonstrate that the model architecture is bounded by the small dataset size for this task over finding an appropriate set of model parameter regularisation and common or basic dataset augmentation techniques. Furthermore, using the base model configuration, we report a new maximum top-1 classification accuracy of 84% on 100 signs, thereby improving on the previous benchmark result for this model architecture and dataset.

## 1. Introduction

Evaluating deep learning models is essential for understanding the limitations of their performance. It is well known that neural networks, and to a greater extent deep neural networks, can require vast amounts of data to effectively learn a model of a system [1]. Along with increasing the training dataset size by simply collecting more examples, other techniques are available that can potentially enhance a model’s performance, both during training and inference.

The objective of training deep neural networks for learning classification models is to subsequently perform the classification task on new data, which may also include data that are different or absent from the dataset used to train the network on. During training, a neural network learns a model of a given dataset that is representative of a system, for example, by performing many iterations (epochs), each consisting of a forward pass through a subset of the dataset followed by a backward pass, which uses backpropagation to calculate and propagate the gradients with respect to a loss function back through the network (other regimes exist). This allows the weights and biases of the neurons—known collectively as the learnable parameters—to be updated with the goal of minimising the calculated error (or loss) between the output and ground truth [2]. In contrast to these learnable parameters, there is a set of hyperparameters that are fixed at the time of training, and the values assigned to these can impact on the performance of a model, not least by also determining the number of learnable parameters. Hyperparameter tuning is the process of finding values for the hyperparameters that optimise the model [3].

Deep neural networks of sufficient complexity, when trained using supervised learning, are subject to the problem of overfitting, by which they effectively begin to memorise the data they learn from. As a rule, a model that overfits a given dataset is less able to generalise well to unseen data [4]. Combatting dataset sensitivity to overfitting can be achieved through applying regularisation techniques such as model parameter regularisation (e.g., [5,6,7]) and data augmentation [8]. Regularisation techniques invariably have one or more associated tunable hyperparameters (including the choice of whether to use a given technique during training), and correctly tuning these hyperparameters is key to optimising the performance of deep learning models [9], particularly when datasets are size limited.

Bergstra and Bengio [10] showed that not all hyperparameters are equal for all models, and for the efficiency of hyperparameter optimisation, they demonstrate both empirically and theoretically that random search is a superior strategy over systematic grid or manual search for finding suitable hyperparameter values, and at a fraction of computational cost. Random search has since become an increasingly popular method for hyperparameter tuning, with modern machine learning platforms providing functionality to automate this process, including quasi-random search techniques for combining a multitude of different hyperparameter values, e.g., W&B Sweep [11]. While random search increases the speed at which optimal (or near-optimal) combinations of hyperparameters can be found—which can be beneficial when there is, for example, a race to market—it does so largely at the expense of understanding the impact individual hyperparameters have on model performance. Traditional ablation studies, for example with manual hyperparameter selection, retain the advantage of being able to isolate any measurable contribution—positive or negative—from each modified hyperparameter, including combinations of hyperparameters albeit at significantly increased computational cost [10].

In this study, we measure the effect each hyperparameter, from a chosen set, has on the model performance of an encoder-only transformer, with a view to further optimise the task of classifying sets of isolated, dynamic signs from human pose estimation keypoints. To this end, we perform a large-scale ablation study in the form of a systematic manual search over select hyperparameter intervals, as well as introducing further regularisation techniques, such as the shrinkage methods: Lasso ($\ell_{1}$ norm) [12] and Ridge ($\ell_{2}$ norm) [13] parameter regularisation. We also apply augmentation techniques on our chosen sign language recognition dataset, which is small by deep learning standards. In addition to common augmentation techniques that include rotating, adding noise, and scaling the keypoints, we also introduce variability by manipulating the frames in the sequences in several ways. We extend the original benchmark study by Woods and Rana [14], which was otherwise limited to an architecture search in the form of the number of transformer encoder layers and attention heads, by substantially increasing the hyperparameter search space as well as doubling the number of experiments conducted per configuration grouping.

Regularisation techniques used in deep learning tasks—in our case performing sign language recognition—take several forms. For convenience, we can split these into two distinct groups: model parameter regularisation, and data augmentation. Both groups have associated hyperparameters that may require tuning; however, some hyperparameters have what are deemed good default values, which have been discovered through previous research, and generally do not require any further tuning. We describe typical model parameter regularisation and data augmentation techniques below.

### 1.1. Model Parameter Regularisation

Model parameter regularisation encompasses both the number of learnable parameters through architectural aspects of a neural network, and restrictions imposed upon those learnable parameters. Regularising model parameters across the entire neural network can help mitigate overfitting by applying penalties during training that encourage learning a less complex model, for example, through having fewer learnable parameters, or by increasing the sparsity of those parameters by promoting fewer neuron activations [15]. This can be thought of as training a neural network that models more of the broad characteristics rather than overly specific nuances of the dataset. Typically, if the model is too complex, it will begin to learn the noise implicit in the training dataset split to the detriment of generalised performance. The purpose of regularisation is to help achieve a balance between learning to model a dataset well while gaining—and subsequently retaining—the ability to make accurate predictions on unseen data that it cannot learn from. Here, we outline the hyperparameters associated with typical model parameter regularisation techniques.

The learning rate dictates how quickly the parameters are updated during optimisation, i.e., the backward pass, which in turn is controlled by a chosen optimisation algorithm, e.g., Adam [16] or Stochastic Gradient Descent [17], each having their own respective hyperparameters. With the addition of a learning rate scheduler (or multiple), of which there are many to choose from, the learning rate can evolve during training based on conditions that include the epoch number or the gradient of the loss value, and so on. A greater learning rate can increase the speed of convergence by taking larger steps towards an optimal configuration, but it can also reduce the potential to achieve an optimised set of parameters by effectively overshooting those that would otherwise most effectively minimise the loss. Conversely, too small a learning rate can lead to a much longer time to achieve convergence, which risks underfitting in the case where the maximum number of epochs is fixed and too small, but also increases the probability of settling on a local minimum in the loss landscape [18]. Reducing the learning rate according to predefined rules is common for converging on optimal model parameters, but many learning rate schedules exist [19].

Batch size is another hyperparameter that can have an effect on model performance [20]. While it is typical to fix the batch size during training, it could in principle, as per the learning rate, also evolve according to other conditions, likely set by more hyperparameters—although this is generally not seen in practice. The type of batching can also be set as a hyperparameter. Taking the entire dataset split in a single batch can speed up convergence and provide more stable parameter updates, but it is more common to use mini-batching, especially in deep learning where datasets are often too big to hold in memory, which is also known to improve generalisation [21]. A mini-batch variation is so-called random batching, where batches are created from randomly selected examples in a dataset split, only completing an epoch when every example has been seen at least once [14].

The number of epochs a neural network is trained for is also tunable; too few can lead to underfitting, where the model is yet to achieve somewhere near-optimal performance on the training dataset split, and too many can lead to overfitting, where further training impairs generalised inference—both of which are undesirable outcomes. Early stopping, however, can provide some protection against overfitting caused by training for too many epochs. Again, this can come with associated hyperparameters, including a patience value or a minimum improvement in some performance metric, like loss or accuracy, before training is stopped. Taking model snapshots when a given metric is improved upon, again possibly determined by a configured hyperparameter, can ameliorate the problems caused by training for too long. Deciding which method works best can depend on the deep learning task, or even be discovered by trial and error.

The number of hidden layers, e.g., in a fully connected neural network, is another tunable hyperparameter. Increased depth, along with choice of activation functions to introduce non-linearities to the model (e.g., ReLU [22]), can enable more complex features to be modelled, but too many hidden layers can contribute to overfitting. Likewise, the number of neurons in a given layer is configurable and has a similar effect in regard to modelling more complex relations between inputs. Increasing the number of neurons can expand the feature space, whereas reducing the number of neurons—as per the encoder part of an autoencoder [23]—can encourage a neural network to learn only the most important features of a model, which can help avoid modelling the training dataset too closely, to reduce overfitting.

Dropout is a technique that randomly sets a fraction of neuron activations output by a given layer to zero, as set by a hyperparameter [24]. Dropout can promote learning redundant and more robust representations that do not rely heavily on individual or specific combinations of neuron activations, which can introduce a form of ensembling by effectively training multiple sub-networks within the neural network to which it is applied. The desired outcome is to regularise the network, so it is less able to learn a perfect model of the training dataset. As with many hyperparameters, the appropriate dropout probability is typically derived empirically [25].

Other techniques include batch normalisation, which ensures the activations of a given layer have zero mean and unit variance to enhance training stability [26], and label smoothing, which can reduce overconfidence in classification predictions [27]. Both of these techniques can help regularise a model to reduce overfitting.

Hyperparameters can also be architecture specific. For example, a convolutional neural network comprises many elements that include the number of convolutional layers, the number, size, and shape of the filters (kernels), the stride and padding values, and so on [28], all of which can be tied to hyperparameters so the optimal combination can be searched for. Likewise, as demonstrated in our parent study [14], a transformer encoder can have hyperparameters that determine the number of layers and attention heads, as well as the number of neurons in the fully connected layers, among others.

### 1.2. Data Augmentation

In supervised deep learning, it is common practice to artificially increase the size of a given dataset by applying data augmentation techniques [29]. This is perhaps most frequently utilised when training neural networks for image processing [30,31], but is also applied to other domains that include natural language processing (NLP) [32], acoustic modelling [33], time-series classification [34], and relation classification [35]. When a single labelled example is augmented, it is modified such that it produces one or more slightly different yet label-preserving, representative examples. Alternatively, new examples can be generated synthetically to also meet the criteria for a given label [36,37]. When applied appropriately, data augmentation is a powerful tool used to increase model robustness to variations and help reduce overfitting. Typical augmentation transformations applied to image data include rotating, scaling, flipping horizontally and vertically, skewing, warping, colour alteration (contrast, hue, temperature, etc.), erasing parts of images, random cropping, injecting noise, and even applying neural style transfer [38], to name several. In our case, we artificially augment our chosen sign language dataset, which consists of human pose estimation keypoints, by applying a limited number of suitable transformations for the domain to examples from the training dataset split only.

It is possible for every data augmentation transformation to be controlled by at least one hyperparameter. For example, the range of angles by which images are rotated would be assigned a respective hyperparameter, as would the range of scaling that was to be applied. In addition to these hyperparameters, there exist more hyperparameters related to which data augmentation methods to use and when, including the mixing probabilities between raw and augmented data when creating batches during training. It is evident that the combination of model parameter regularisation and data augmentation leads to a hyperparameter space that has the potential to be extremely large for a given neural network, and finding optimal hyperparameter values can be a challenging task.

Beyond data augmentation, it is common practice to normalise the input values as a pre-processing step, which can come before and/or after augmentation. Normalisation can take many forms and is used to standardise a dataset to both help and increase the speed of model convergence [39].

### 1.3. Related Work

We now consider related work and the variety of techniques utilised to regularise models for sign language recognition from published research that makes use of the same base dataset of keypoints.

For their SPOTER model, Bohacek and Hruz [40] apply randomised data augmentations to the keypoints during training, with the probability of an augmentation being applied set to 0.5. These augmentations include rotating all keypoints about the centre point by a randomly selected angle in the interval [−13,13); two types of perspective transformations, which are performed on all keypoints excluding those for the hands; sequential rotation of the arm keypoints; and adding Gaussian noise. The first transformation uniformly squeezes every frame of a given sequence by up to 15% of the original frame width, and the second performs a perspective transformation ostensibly to simulate the effect of camera tilt with respect to the subject. The sequential rotation of the arm joints is intended to mimic slight variances in a given sign, which the authors claim do not change the meaning of the signs. They subsequently normalise by scaling all keypoints (treating the hands and body keypoints separately) to lie in the interval $\left[0,1\right]$ before shifting by −0.5 in the *x* and *y* planes. The authors fix the seeds of the random number generators, and as such, their training regime does not assess the impact random seed initialisation has on the variability of model performance. In addition, they do not utilise a learning rate scheduler, nor do they use weight decay with their chosen stochastic gradient descent optimiser. Their transformer model architecture is fixed at 6 encoder and decoder layers, each having 9 attention heads, with a hidden layer dimension of 108 neurons and feed-forward layers with 2048 neurons. They use a learned positional encoding for the encoder input, and a class query for the modified decoder input.

Following SPOTER [40], Eunice et al.’s [41] Sign2Pose model appears to have an almost identical architecture. There are some minor differences, which include slight variations to the augmentations applied (e.g., rotations being expanded from randomly selected angles in [−13,13) in SPOTER to [−15,15) in Sign2Pose) and the inclusion of weight decay with the $\ell_{2}$ penalty hyperparameter set to $10^{-4}$. There is an additional augmentation transformation not performed in the SPOTER study, however, in that keypoints are flipped horizontally with a probability of 0.5, which would be problematic for the signs left and right if they are present in the dataset splits used. Another minor difference is the number of so-called head units chosen to define the signing space, although the underlying mechanism is the same. The one striking difference, however, is the method by which frames that are deemed to be redundant are discarded, which appears to have a significant impact on model accuracy.

For their Pose-GRU model, Li et al. [42] use 2 stacked gated recurrent unit (GRU) layers, regularised by configuring each GRU’s hidden layers with dimensions of 64, 64, 128, 128, which they claim to derive empirically, and do not alter throughout. They randomly select 50 frames from each example sequence, which provides some augmentation through randomisation, and perform classification on every frame within a sequence, including the output pooling, to derive a final prediction. They use cross-entropy loss and the Adam optimiser. In contrast, their graph convolution network (GCN) based model, Pose-TGCN, appears to apply no explicit regularisation techniques other than early stopping, which they apply to all training runs, stopping when the validation accuracy no longer increases or the number of elapsed epochs reaches 200.

Tunga et al. [43] appear to extend Pose-TGCN by connecting it to a BERT transformer [44], which they refer to as GCN-BERT. They also use cross-entropy loss and the Adam optimiser, regularised with the weight decay $\ell_{2}$ penalty hyperparameter set to $10^{-8}$, and train for 100 epochs. No other details are provided that document any other techniques employed to improve model performance.

### 1.4. Contributions

This article presents the first comprehensive, large-scale ablation study of an encoder-only transformer for modelling sign language using human pose estimation keypoint data. We quantify the effects that a variety of regularisation and augmentation techniques have on model performance. We show that fixing the random number generator seeds and repeating a single experiment with a hyperparameter value change is an inadequate method for universally determining the outcome of that hyperparameter change. We also, in the case of this task and dataset consisting of sparse values, provide strong evidence to support the hypothesis that the size of the dataset is the limiting factor beyond any of the regularisation or augmentation techniques applied for improving the performance of our model to classify isolated, dynamic signs.

### 1.5. Article Organisation

The remainder of this article is organised as follows: Section 2 details the materials and methods used in this study, including comprehensive descriptions of the regularisation and augmentation techniques applied; Section 3 lists the outcome of every experiment conducted; Section 4 gives in-depth discussion and analysis of the results; and finally, Section 5 provides conclusions to be drawn from the study.

## 2. Materials and Methods

### 2.1. Dataset

Following the benchmark established by Woods and Rana [14], we conduct our experiments on a significantly improved version of WLASL [42], called WLASL-alt [45]. This release has corrections applied to the original WLASL dataset that include incorrect sign labels and typing errors, as discussed by Neidle and Ballard [45], and Dafnis et al. [46]. The underlying data in WLASL consist of human pose estimation keypoints, extracted using OpenPose [47]. Each keypoint relates to a landmark on the human body that has been extracted from the frames that make up isolated dynamic sign sequences, and is defined by three values: *x*- and *y*-axis coordinates, and a detection confidence score (which we do not use). We normalise the keypoint coordinate values as per the original study [14], which scales all keypoints in a given sequence such that the mean *x*-axis distance between the shoulder keypoints on every frame of that respective sequence is unity, and likewise for the mean *y*-axis distance between the neck and nose keypoints. The train, validation, and test dataset splits used match the original study, and are available online at https://github.com/ltwoods/msl (accessed on 27 August 2023).

### 2.2. Experimental Setup

The HILDA HPC at DARTeC [48] was used for all experiments, which hosts a virtual machine (VM) running Ubuntu Linux 20.04.4 LTS with a 5.15.x series kernel, an Intel Xeon Gold 6258R with 112 CPUs, 377 GiB RAM, and 4 NVIDIA A100 40 GiB GPUs, with Python 3.8.10, PyTorch 1.9.0+cu111, and NumPy 1.21.4.

### 2.3. Experiments

Except where stated otherwise, we train for 200 epochs, taking model snapshots once per epoch when a performance metric is improved upon on the validation dataset split, and testing with the best model snapshot from the corresponding training run. Using both increased validation set accuracy and reduced validation set cross-entropy loss as performance metrics, Woods and Rana [14] showed the difference in test set accuracy between the two best models was negligible for this task. To reduce the number of similar results reported, we therefore choose to report results from the loss performance metric model only. This choice is further justified because cross-entropy provides a theoretically better measure of the overall predictability of a model compared to a single real-valued metric based only on the number of correctly predicted class labels, as would be the case with only using the improved accuracy score per epoch—we acknowledge, however, that in practice, the difference between the two sets of experiments is negligible.

We perform all experiments on the same 100 sign classes, which we choose with the intention of providing a reasonable balance between the time taken to perform each experiment and the complexity of the experiment (i.e., number of signs to classify). The sizes of the dataset splits are detailed in Table 1. We also perform all experiments with a single encoder layer and four attention heads. This combination of encoder architecture and testing with the best-performing model based on loss correlates with the best-performing configuration for 100 classes from the original study [14]. We run experiments to test the classification performance impact from changes to the way the neural network is trained, which includes the way batches are created during each epoch and explicit regularisation techniques. In contrast to the original study, we increase the number of experiments per configuration from 8 to 16, with the aim of reducing the experimental uncertainty of observations and help identify smaller changes in mean performance. In every experiment group, we include the result from the baseline experiment as the control.

Where appropriate, we choose hyperparameter values that step up in intervals as select proportional powers of two, e.g., batch size $\lambda_{\mathrm{batch}}=2^{n}\;\forall n\in \left[4,\ldots ,11\right]$, or added noise $\lambda_{\mathrm{noise}}=0.001\times 2^{n}\;\forall n\in \left[0,\ldots ,9\right]$. The intention is to strike a balance between measuring the effects of smaller changes, as well as the effects of much larger changes, per experiment group. In other cases, we increment linearly, e.g., encoder dropout $\lambda_{\mathrm{encdo}}=0.1\times n\;\forall n\in \left[1,\ldots ,8\right]$.

While extensive, the experiments we conduct are not exhaustive, and given the available resources, we limit ourselves to the groups performed in this ablation study, which are detailed below. Unless specified otherwise, all experiments use the baseline parameters listed in Table 2. In each experiment group, we only modify the hyperparameter to be studied while keeping all other values constant according to the baseline configuration. Parameters that are left at their default implementation values are omitted, for example, all of the CrossEntropyLoss arguments in the PyTorch implementation [49].

Where appropriate, we analyse the results using a suitable statistical test. For example, in the case of determining the statistical significance of a given hyperparameter change, we use the independent two-sample *t*-test with the assumption that our data are normally distributed and of equal variance. The independent two-sample *t*-test produces a probability value, *p*, and a test statistic, *t*, which is calculated as

$$t=\frac{{\bar{x}}_{1}-{\bar{x}}_{2}}{s_{p}\sqrt{\frac{1}{n_{1}}+\frac{1}{n_{2}}}},$$

with the pooled standard deviation, $s_{p}$, calculated as

$$s_{p}=\sqrt{\frac{\left(n_{1}-1\right)s_{1}^{2}+\left(n_{2}-1\right)s_{2}^{2}}{n_{1}+n_{2}-2}},$$

where ${\bar{x}}_{1}$ and ${\bar{x}}_{2}$ are the mean values of the respective samples, $n_{1}$ and $n_{2}$ are the sample sizes, and $s_{1}$ and $s_{2}$ are the standard deviations of the samples. In our case, the sample sizes $n_{1}$ and $n_{2}$ are equal. Throughout, we adopt a significance level α=0.05 for our statistical tests. We acknowledge that at 16 experiments per group, our sample size is relatively small.

#### 2.3.1. Random Batching Versus Single Pass

We compare the outcome between randomly selecting batches of sequences with a single pass through the respective dataset split. Random batching is a mini-batching technique, where batches of data are selected from a given dataset split on a random basis, and an epoch only completes when every data point has been seen at least once. In contrast, a single pass through the dataset has each batch consist of a fixed-size and ordered slice of a dataset split. It is common to discard any dataset points that do not fit into a batch, i.e., dmodb>0, where *d* is the dataset split size and *b* is the batch size. In our case, however, we have a small dataset and so when using the single-pass method, we automatically reduce the size of the batch accordingly when batching the last sequences in a dataset split to ensure we use all available data. Experiments that use single pass are slower to converge in terms of epochs completed—which we otherwise fix to 200 for all experiments that use random batching—so we measure the outcome across a range of maximum epochs per training run. The number of epochs, $\lambda_{\mathrm{epochs}}$, for single-pass experiments are

$$\lambda_{\mathrm{epochs}}=\left\{200,500,1000,1500\right\}.$$

#### 2.3.2. Batch Size

We measure the effect batch size, $\lambda_{\mathrm{batch}}$, has on outcome by training over batch sizes

$$\lambda_{\mathrm{batch}}=\left\{16,32,64,128,256,512,1024,2048\right\}.$$

#### 2.3.3. Learning Rate

We measure the effect learning rate, $\lambda_{\mathrm{lr}}$, has on outcome by training with the values

$$\lambda_{\mathrm{lr}}=\left\{0.00001,0.00005,0.0001,0.0005,0.001,0.005,0.01,0.05\right\}.$$

#### 2.3.4. $\ell_{1}$ and $\ell_{2}$ Parameter Regularisation

We apply $\ell_{1}$ [12] and $\ell_{2}$ [13] parameter regularisation to measure the effect each has on reducing overfitting, and subsequently on test set accuracy. Both forms impose a penalty on the loss proportional to the sum or square of the sum, respectively, of the model weights. $\ell_{1}$ parameter regularisation promotes sparsity in neuron activations by effectively pushing some weights to zero, whereas $\ell_{2}$ parameter regularisation encourages smaller weights throughout the network, thereby decreasing the probability that particular neurons, and by extension selected features, dominate the learning process. Fewer activations produce a simpler model, which can help feature selection, and in turn help reduce overfitting, as can a more evenly distributed contribution from activations across the neurons. More formally, given *N* model weights, we apply $\ell_{1}$ to the batch loss, $L_{\mathrm{batch}}$, as

$$L_{\mathrm{batch}}=E\left(y,\hat{y}\right)+\lambda_{\ell_{1}}\sum_{n=1}^{N}\left|w_{n}\right|,$$

and $\ell_{2}$ as

$$L_{\mathrm{batch}}=E\left(y,\hat{y}\right)+\lambda_{\ell_{2}}\sum_{n=1}^{N}w_{n}^{2},$$

where $E\left(y,\hat{y}\right)$ is the error calculated by the loss function given the ground-truth label *y* and prediction $\hat{y}$, and $\lambda_{\ell_{1}}$ and $\lambda_{\ell_{2}}$ are the associated parameter regularisation hyperparameters. We test the effect $\ell_{1}$ has over the values

$$\lambda_{\ell_{1}}=\left\{0.00001,0.00005,0.0001,0.0005,0.001,0.005,0.01,0.05,0.1\right\}.$$

and likewise for $\ell_{2}$ over

$$\lambda_{\ell_{2}}=\left\{0.00001,0.00005,0.0001,0.0005,0.001,0.005,0.01,0.05,0.1\right\}.$$

Because $\ell_{2}$ parameter regularisation is implemented by the Adam optimiser through the weight decay parameter [50], we use $\ell_{2}$ parameter regularisation by default in every experiment that uses the common baseline parameters. Experiments that also apply $\ell_{1}$ parameter regularisation do so in a so-called elastic net regularisation configuration [51] with the parameter $\lambda_{\ell_{2}}$ held constant throughout at $\lambda_{\ell_{2}}=0.001$. We do not test elastic net regularisation configurations with other values of $\lambda_{\ell_{2}}$.

#### 2.3.5. Encoder Feed-Forward Block Layer Dimension

Within the encoder, the attention block is followed by a feed-forward block. The first component of this is a position-wise fully connected feed-forward layer, which has a configurable output dimension. This is followed by an activation function, after which dropout is applied. Another feed-forward layer follows, which has the same configurable input dimension as the first layer’s output dimension. It is, therefore, this shared dimension that we refer to when we mention the feed-forward block layer dimension. We measure the effect that creating a bottle-neck or expanding the feature space between these feed-forward layers has, compared with keeping it constant, by setting the dimension, $\lambda_{\mathrm{ff}}$, to

$$\lambda_{\mathrm{ff}}=\left\{2,4,8,16,32,64,108,128,216,256,512,1024,2048,4096,8192\right\}.$$

In addition to setting the layer dimension to typical powers-of-two values, we also keep it constant at $\lambda_{\mathrm{ff}}=108$, as well as doubling it at $\lambda_{\mathrm{ff}}=216$.

#### 2.3.6. Encoder Dropout

Within the encoder, dropout is applied in both the self-attention block and the feed-forward block with the same probability, $\lambda_{\mathrm{encdo}}$, which we also measure by varying over the values

$$\lambda_{\mathrm{encdo}}=\left\{0.0,0.1,0.2,0.3,0.4,0.5,0.6,0.7,0.8\right\}.$$

#### 2.3.7. Embedding Dropout

Likewise, we also apply dropout across a range of probabilities to the keypoint embeddings and measure by varying the associated hyperparameter, $\lambda_{\mathrm{embdo}}$, over the values

$$\lambda_{\mathrm{embdo}}=\left\{0.0,0.1,0.2,0.3,0.4,0.5,0.6,0.7,0.8\right\}.$$

#### 2.3.8. Augmentation

Augmentation is the act of manipulating the training dataset split to provide the model with extra examples that vary slightly, effectively extending the dataset. We apply augmentation in several ways and measure the effect of each augmentation type, with each having an associated hyperparameter acting as an upper limit to the amount of augmentation applied, e.g., a rotation augmentation specified by $\lambda_{\mathrm{rot}}=10$ randomly selects a rotation value in the interval $\left[0,10\right]$. Hence, when enabled, we apply a given augmentation to every batch because this also includes batches that receive no effective augmentation, e.g., the rotation randomly selected between zero and the maximum value happens to be zero. We acknowledge the potential power of so-called sequential rotation as an augmentation technique [40,41], but we choose not to apply this or similar augmentation techniques to avoid inadvertently altering any of the signs beyond their original meaning. As such, we do not anticipate that our augmentations alter the meaning of any signs. In all cases where the respective augmentation parameter is zero, we use a common group of results from the base model configuration as the control.

We inject varying amounts of noise into the *x*- and *y*-coordinate values of each keypoint. Using a configuration hyperparameter $\lambda_{\mathrm{noise}}$, we generate a random value per keypoint coordinate, per frame, per sequence in the batch, in the interval $\left[-\lambda_{\mathrm{noise}},\lambda_{\mathrm{noise}}\right)$ using NumPy’s uniform [52]. We generate maximum positive and negative amounts of noise using the values

$$\lambda_{\mathrm{noise}}=\left\{0.000,0.001,0.002,0.004,0.008,0.016,0.032,0.064,0.128,0.256,0.512\right\}.$$

We rotate the keypoint coordinate values with the intention of introducing slight variations in camera orientation about the axis that goes into the scene. Per batch, we rotate the keypoints of every frame in all sequences about the origin by the same value in degrees specified by the maximum rotation hyperparameter $\lambda_{\mathrm{rot}}$, which is randomly generated—again using uniform—to be in the interval $\left[-\lambda_{\mathrm{rot}},\lambda_{\mathrm{rot}}\right)$, using the values

$$\lambda_{\mathrm{rot}}=\left\{0,1,2,4,8,16,32\right\}.$$

We apply three different forms of scaling. We scale along each of the *x*- and *y*-axes separately, as well as both simultaneously, by a scaling factor that is determined by a common hyperparameter $\lambda_{\mathrm{scale}}$. Again using uniform, we randomly generate a scaling factor in the interval $\left[1.0-\lambda_{\mathrm{scale}},1.0+\lambda_{\mathrm{scale}}\right)$ and apply this to every *x*- and *y*-axis coordinate in a given batch, as configured, e.g., if scale augmentation is configured for the *x*-axis only, the scaling factor is applied to the *x*-axis coordinate values. We scale using the maximum values

$$\lambda_{\mathrm{scale}}=\left\{0.00,0.01,0.02,0.04,0.08,0.16,0.32\right\}.$$

To simulate the subtle effect of increased signing speed in various places, we drop frames from a given sequence and pad the rest of the sequence with copies of the last frame. Using the hyperparameter $\lambda_{\mathrm{drop}}$, we select a random sample of frames to remove from every example in a given batch using the values

$$\lambda_{\mathrm{drop}}=\left\{0,1,2,4,8,16,32\right\}.$$

Example sequences can have a period of effective silence before a given sign begins. We simulate the effect of signs beginning at slightly different times by trimming a number of frames from the beginning of a sequence as dictated by the hyperparameter $\lambda_{\mathrm{trim}}$. As with the drop frames augmentation, each sequence in a given batch is padded with the data from the respective sequence’s last frame. The random number of frames to trim from the start lies in the interval $\left[1,\lambda_{\mathrm{trim}}\right]$ and is randomly generated from the values

$$\lambda_{\mathrm{trim}}=\left\{0,1,2,4,8,16,32\right\}.$$

Offsetting frames in a given batch of sequences intends to have the opposite effect of trimming the start. This simulates each sign sequence effectively beginning later than the original signs in those sequences. We do this by taking a random number of copies of the first frames as per the hyperparameter $\lambda_{\mathrm{ocopy}}$, and inserting them at the beginning of each sequence of a batch. The same number of frames from the end of the sequences are removed to maintain the fixed sequence lengths. We cap the number of frames to copy to prevent overflow on the sequence length in the event a sequence is shorter than the number of frames selected to insert. The frames to copy are randomly generated in the interval $\left[1,\lambda_{\mathrm{ocopy}}\right]$ from the values

$$\lambda_{\mathrm{ocopy}}=\left\{0,1,2,4,8,16,32\right\}.$$

As with the offset frames copy augmentation, we also apply an augmentation by inserting empty frames in place of copies of the first frame of the sequences in a given batch as determined by the hyperparameter $\lambda_{\mathrm{opad}}$, again randomly generated to be in the interval $\left[1,\lambda_{\mathrm{opad}}\right]$ from the values

$$\lambda_{\mathrm{opad}}=\left\{0,1,2,4,8,16,32\right\}.$$

#### 2.3.9. Fixed-Seed Comparison on Singular Hyperparameters

It is common for random number generator seeds to be fixed to enable repeatability of experiments (on a given computer). The implied assumption is that changes to a single hyperparameter for a common fixed-seed value across experiments will reveal the explicit impact that hyperparameter has on outcome. We test this by running 8 baseline experiments with a fixed-seed set by the hyperparameter $\lambda_{\mathrm{seed}}=\left\{0,1,2,3,4,5,6,7\right\}$, and repeating experiments with singularly altered hyperparameters using the same seeds. We measure the outcome of experiments with fixed seeds for the following hyperparameter settings: $\lambda_{\mathrm{ff}}=\left\{108,2048\right\}$, $\lambda_{\mathrm{rot}}=\left\{0,20\right\}$, $\lambda_{\mathrm{scale}}=\left\{0,0.08\right\}$, and $\lambda_{\mathrm{encdo}}=\left\{0.0,0.3\right\}$, where the first value in each hyperparameter set is the control.

#### 2.3.10. Fixed-Seed Comparison on Normalisation

Normalising data has been shown to work effectively for this task [14], and augmentations that alter the position of keypoint coordinates or the frames within a sequence can, in principle, alter the distribution of keypoint values across a sequence to the detriment of a neural network’s ability to efficiently learn a model of this system. Although the effect is anticipated to be negligible, we nevertheless also compare two groups of 8 experiments; with and without renormalising after augmentation, separately, with hyperparameters $\lambda_{\mathrm{rot}}=20$, and $\lambda_{\mathrm{scale}}=0.08$ applied to both the *x*- and *y*-axes. As with the experiment in Section 2.3.9, the same fixed seed is given to each of the paired with-and-without renormalisation experiments. Repeating an experiment on the same computer with the same configuration and fixed seed produces the same result, so the intention is to test the impact renormalisation has on model performance after altering the keypoint distribution through augmentation and analyse the distribution of results (rather than performing a one-to-one comparison).

#### 2.3.11. Dataset Size

We reduce the dataset size to measure model performance as a function of the total number of examples. We do this by uniformly reducing the number of examples in each class by a configured ratio, $\lambda_{\mathrm{red}}$. This does not fix the class imbalance problem, which is a known characteristic of this dataset [14,46], but it does linearly scale the size by keeping the ratios as constant as possible. We measure the reduction in dataset size for the values

$$\lambda_{\mathrm{red}}=\left\{0.0,0.1,0.2,0.3,0.4,0.5,0.6\right\}.$$

## 3. Results

Following Woods and Rana [14], all quoted experimental uncertainties are calculated as $\pm 1.96\times \sigma_{M}$, where $\sigma_{M}=\frac{\sigma }{\sqrt{n}}$, and σ is the standard error of the mean over *n* repeated experiments. All results report the mean top-1 classification accuracy, for the same 100 classes, using the best-performing models during training as measured by the loss performance metric per experiment. The highest accuracy values in each results table are highlighted in bold.

First, we report the results from experiments that compare random batching against a single pass through the dataset per epoch, which are shown in Table 3. Random batching results are from a single group of 16 experiments over 200 epochs, while single-pass experiments are groups of 16 experiments repeated over a number of epoch ranges.

Table 4 shows the results from varying the batch size using random batching across a typical range of batch sizes.

Table 5 shows the results from varying the learning rate. All experiments use the same cosine annealing with warm restarts learning rate scheduler.

We now report the results from applying $\ell_{1}$ and $\ell_{2}$ parameter regularisation in Table 6. As described in Section 2.3.4, all experiments where $\ell_{1}$ parameter regularisation is applied also have $\ell_{2}$ parameter regularisation applied because it is implemented as weight decay in the Adam optimiser. For these experiments, the value of $\ell_{2}$ parameter regularisation is kept constant throughout. Experiments where either $\ell_{1}$ or $\ell_{2}$ parameter regularisation is not applied are marked with –.

The results from varying the number of neurons in the encoder feed-forward block are listed in Table 7.

Table 8 shows the results from incrementing the encoder dropout probability from $\lambda_{\mathrm{encdo}}=0.0$ to $\lambda_{\mathrm{encdo}}=0.8$.

Likewise, the results from incrementing the input embedding dropout probability from $\lambda_{\mathrm{embdo}}=0.0$ to $\lambda_{\mathrm{embdo}}=0.8$ are listed in Table 9.

We now report the results from augmenting the training dataset split. Table 10 shows the results from augmenting the input data with added noise.

Table 11 shows the results from augmenting the input data with added rotation about the chosen origin.

Table 12 shows the results from augmenting the input data by scaling along the *x*- and *y*-axes.

We report the results from augmenting the input data by dropping random frames from every sequence in a batch, up to a configured maximum value. Table 13 shows the mean top-1 test accuracy results for 100 classes.

Table 14 shows the results from augmenting the input data by trimming a random number of frames from the start of every sequence in a batch, up to a configured maximum value.

The results from augmenting the input data by offsetting the beginning of each sequence in a batch by inserting a random number of copies of the first frame at the start, up to a configured maximum value, are shown in Table 15. We remove the corresponding number of frames from the end of the sequences to keep their lengths unchanged.

Similarly, the results from augmenting the input data by offsetting the beginning of each sequence in a batch by inserting a random number of blank frames at the start, up to a configured maximum value, are shown in Table 16. As with the offset frames copy augmentation, we remove the corresponding number of frames from the end of the sequences to keep their lengths unchanged.

We report the results from fixing the random number generator seeds and comparing the outcome on a singular hyperparameter change in Table 17. Running experiments with fixed seeds allows us to ascertain whether the impact a specific hyperparameter has is measurable from a single experiment.

The results from fixing the random number generator seeds and comparing the effect renormalisation after augmentation has on model performance are reported in Table 18.

Finally, we report the results from the dataset size experiment, where we measure the impact dataset size has on model performance by reducing the number of class examples by a configured ratio. These results are presented in Table 19.

## 4. Discussion

**Figure 1:**
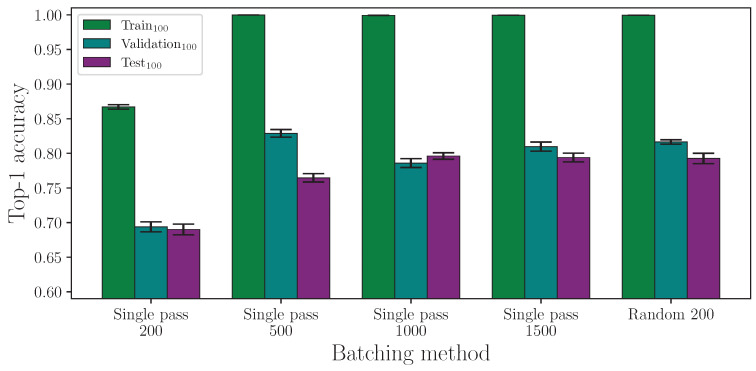
Batching method: single pass versus random batching for top-1 classification accuracy on 100 signs. Each column label includes the number of epochs the neural network was trained for.

We begin by evaluating the two batching methods. As Figure 1 shows, single-pass batching requires many more epochs before achieving a model accuracy that is comparable to random batching, at which point neither method is clearly the more optimal. This is because models trained using single-pass batching underfit the data when trained for an insufficient number of epochs. Woods and Rana [14] showed that, for this task, the majority of models trained using random batching converge at around 130 epochs, and we can expect all models trained using this method to converge by approximately 250 epochs. It is therefore possible that, at 200 epochs, some of the random batching experiments had not fully converged before training had been completed. Nevertheless, given enough iterations through the data for single-pass batching (e.g., ∼1000 epochs), both methods are comparable in terms of performance on the test set. Using the number of epochs as a metric for time to train can be misleading, however, because random batching, in practice, makes multiple iterations through the majority of the entire dataset per epoch, and in doing so, each epoch takes more time to complete than with the single-pass batching method.

Taking the results from single-pass batching at 1500 epochs and comparing with random batching at 200 epochs using an independent two-sample *t*-test, we find the difference between the respective train and validation accuracy result groups are statistically significant at $p=3.255\times 10^{-18}$ and $p=1.851\times 10^{-6}$, respectively, with both mean accuracies being greater on the random batch experiments. However, for the test accuracy, at $p=7.682\times 10^{-1}$, we find no statistical significance between the two experiment groups showing that both methods are comparable in the test set performance. The full top-1 accuracy results for the batching method experiment analysis are listed in Table A1 and Table A2.

**Figure 2:**
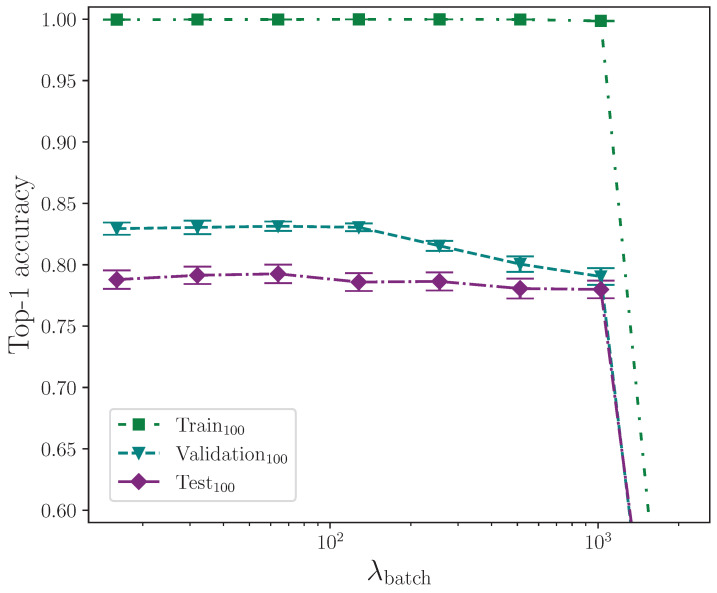
Log-linear plot showing mean top-1 accuracy as a function of batch size, $\lambda_{\mathrm{batch}}$, for 100 classes. Somewhere between a batch size of 1024 and 2048, model performance begins to rapidly degrade.

Comparing the impact of batch size, we observe no appreciable effect on test set accuracy up to at least batch size $\lambda_{\mathrm{batch}}=1024$, after which performance drastically degrades (see Figure 2). We did not test any batch sizes between $\lambda_{\mathrm{batch}}=1024$ and $\lambda_{\mathrm{batch}}=2048$, so we cannot provide a more precise estimate for which model performance begins to degrade. When using random batching, larger batch sizes equate to fewer iterations through the data, which explains the observed reduction in accuracy once a threshold is met. As the batch size increases, random batching begins to approximate single pass, which we show to perform worse than random batching at lower epochs. We also observe a reduction in validation set performance for batch sizes $\lambda_{\mathrm{batch}}>128$, indicating some dataset imbalance.

**Figure 3:**
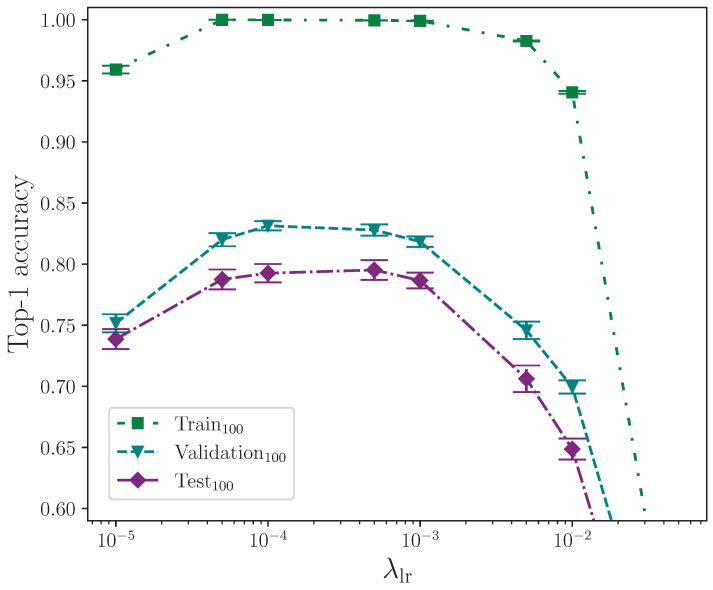
Log-linear plot showing mean top-1 accuracy as a function of learning rate, $\lambda_{\mathrm{lr}}$, for 100 classes. A learning rate between approximately $\lambda_{\mathrm{lr}}=1.0\times 10^{-4}$ and $\lambda_{\mathrm{lr}}=1.0\times 10^{-3}$ produces the best-performing models.

Reviewing the results from the learning rate experiment, we observe that, for this task, the learning rate that produces the best model performance is between approximately $\lambda_{\mathrm{lr}}=1.0\times 10^{-4}$ and $\lambda_{\mathrm{lr}}=1.0\times 10^{-3}$, with a reduction in accuracy outside of that range up to the values measured. Figure 3 clearly identifies the peak performance range of learning rate values, with $\lambda_{\mathrm{lr}}=5.0\times 10^{-4}$ giving the best test set accuracy over the range of learning rates tested.

We analyse the effect that $\ell_{1}$ and $\ell_{2}$ parameter regularisation has on model performance by plotting the outcomes of their respective experiments separately. In both cases, the comparative model performance from applying no parameter regularisation is overlaid, which shows the mean top-1 performance for the train, validation, and test sets as dotted lines, with the respective calculated uncertainty making up the shaded areas. No shaded area is visible for the training set performance because its value is zero when evaluated to five significant figures. For convenience, we limit the *y*-axis range of both plots equally to exclude values that correspond with very low performance outcomes, as we do on other plots where performance is severely reduced as the result of a hyperparameter value.

**Figure 4:**
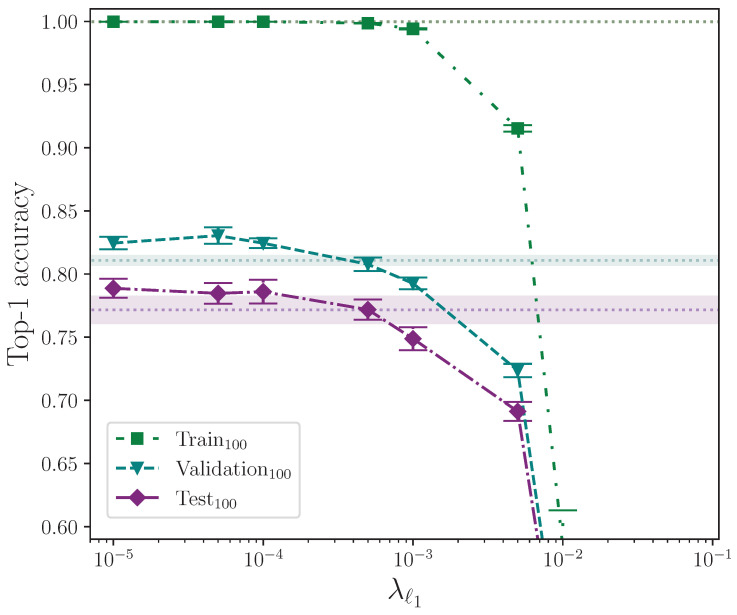
Log-linear plot showing mean top-1 accuracy as a function of elastic net regularisation for 100 classes, with the $\ell_{1}$ parameter, $\lambda_{\ell_{1}}$, varied, and the $\ell_{2}$ parameter, $\lambda_{\ell_{2}}$, held constant at $\lambda_{\ell_{2}}=0.001$. The dashed lines with shaded areas indicate the respective mean top-1 accuracy minimum and maximum values from the calculated uncertainty for 16 experiments with no elastic net regularisation applied at all.

Figure 4 shows the effect of applying elastic net regularisation over a range of values for the $\ell_{1}$ parameter, $\lambda_{\ell_{1}}$, with a fixed $\ell_{2}$ parameter, $\lambda_{\ell_{2}}=0.001$. The shaded areas indicate the mean top-1 accuracy minimum and maximum values from the calculated uncertainty for 16 experiments with no parameter regularisation being applied, which allows the effect of elastic net regularisation to be observed. By also referring to Table 6, it is clear that values of $\lambda_{\ell_{1}}>0.005$ start to significantly negatively impact on model performance. Conversely, values of $\lambda_{\ell_{1}}\leq 0.0001$ show the models perform better than without any parameter regularisation being applied, with a mean top-1 accuracy gain of approximately 1.7% on the test set for the best-performing value $\lambda_{\ell_{1}}=0.00001$. The trend suggests values of $\lambda_{\ell_{1}}<0.00001$ may offer further, albeit marginal, gains, but it must be noted that the error of these measurements does lie within the range of the baseline error, which in this case is a model with no parameter regularisation. At this point in the analysis, therefore, we cannot rule out the possibility that the observed gains are superficial. With the assumption that there is a real performance gain at very low values of $\lambda_{\ell_{1}}$, it appears to be the case that, in conjunction with $\ell_{2}$ parameter regularisation, this technique works best for this task when $\lambda_{\ell_{1}}$ is included somewhat sparingly.

Applying the independent two-sample *t*-test to the results from the experiments with the best-performing value of $\lambda_{\ell_{1}}=0.00001$ and those with no parameter regularisation (the control), we find no significant difference between the two groups for the train accuracy, with $p=4.466\times 10^{-1}$. We do, however, find a significant difference in the validation and test accuracies, with $p=3.149\times 10^{-4}$ and $p=1.966\times 10^{-2}$, respectively, with both group mean values being greater than the control group with no parameter regularisation. This strongly indicates that elastic net regularisation with a very low value for $\ell_{1}$ improves model performance compared with no parameter regularisation. The full top-1 accuracy results for the elastic net regularisation and control experiments analysis are listed in Table A3 and Table A4.

**Figure 5:**
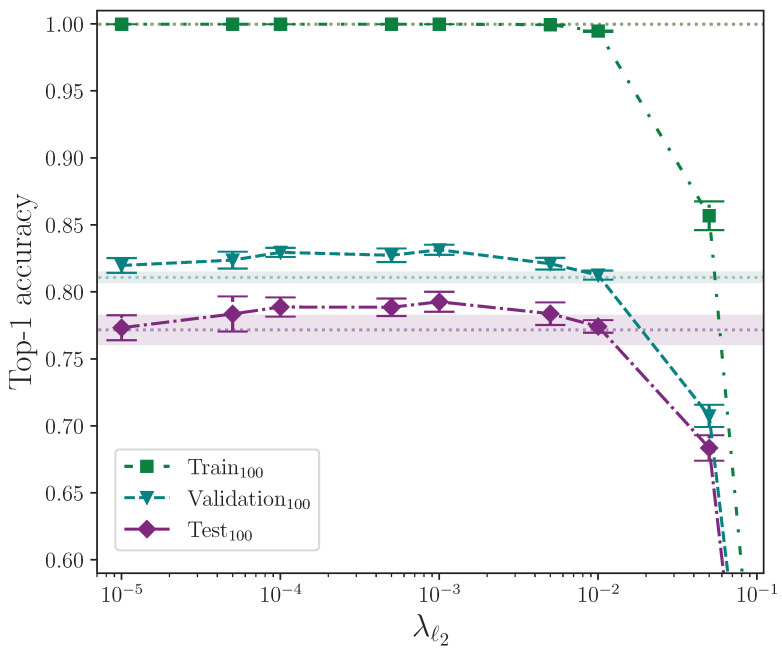
Log-linear plot showing mean top-1 accuracy as a function of $\ell_{2}$ parameter regularisation, $\lambda_{\ell_{2}}$, for 100 classes. The dashed lines with shaded areas indicate the respective mean top-1 accuracy and associated uncertainty for 16 experiments with no parameter regularisation applied.

Figure 5 shows the effect of applying $\ell_{2}$ parameter regularisation alone. As with elastic net parameter regularisation, there is a clear degradxation in model performance for larger related parameter values. The model is, however, less sensitive to larger values of $\lambda_{\ell_{2}}$ when compared with the magnitude of $\lambda_{\ell_{1}}$ because of the definition of the penalty imposed on the neuron weights (see Section 2.3.4). There is a clear measured optimal value at $\lambda_{\ell_{2}}=0.001$, which does not intersect with the baseline error (wherex the baseline, again, is a model with no parameter regularisation). This value of $\lambda_{\ell_{2}}$ provides an observed mean performance gain of approximately 2.1% over no parameter regularisation.

Performing an independent two-sample *t*-test on the results from the experiments with the best-performing value of $\lambda_{\ell_{2}}=0.001$ and those with no parameter regularisation, we find—again, similar to elastic net regularisation—that there is no significant difference between the two groups for the train accuracy, with $p=8.085\times 10^{-1}$, but we do find the difference is significant for the validation and test accuracies, which have $p=1.153\times 10^{-7}$ and $p=5.080\times 10^{-3}$, respectively. In both of these latter cases, the mean values are again greater than the control. We conclude that $\ell_{2}$ parameter regularisation improves model performance by a significant margin.

It would be instructive to perform $\ell_{1}$ parameter regularisation experiments as a standalone group without elastic net regularisation. Despite this, the selected value for the $\ell_{2}$ parameter, $\lambda_{\ell_{2}}=0.001$, can be attributed to being a fortunate choice for the common baseline hyperparameters. The observed best combination of hyperparameters for the elastic net configuration is almost certainly caused by the contribution made by the $\ell_{2}$ component over $\ell_{1}$, and we can test this statistically. This would explain why $\ell_{2}$ parameter regularisation performs better than elastic net regularisation, and why elastic net regularisation performs best for extremely low values of $\lambda_{\ell_{1}}$ where the $\ell_{1}$ contribution is significantly reduced. Comparing the outcome of the statistical tests performed on the elastic net and $\ell_{2}$ parameter regularisation, with $p=1.966\times 10^{-2}$ and $p=5.080\times 10^{-3}$, respectively, we observe that, statistically, $\ell_{2}$ parameter regularisation alone produces a more performant model on the test set, although experimenting with different combinations of hyperparameter values would provide more confidence. The full top-1 accuracy results for the $\ell_{2}$ parameter regularisation and control experiments analysis are listed in Table A3 and Table A5.

**Figure 6:**
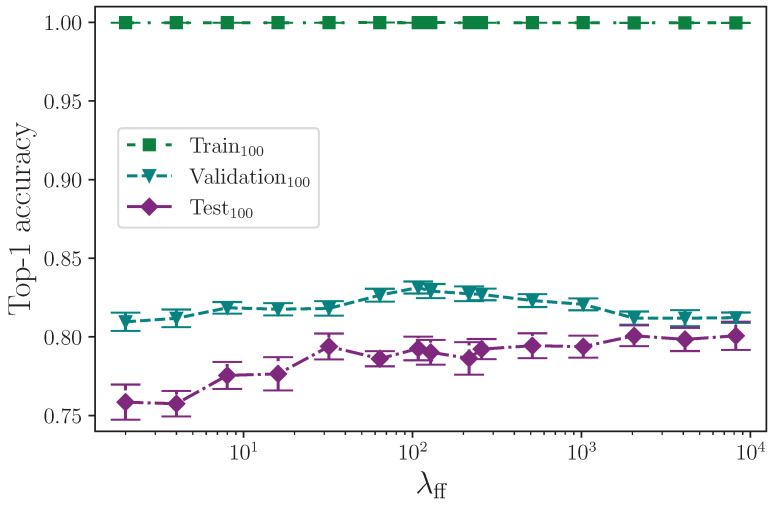
Log-linear plot showing mean top-1 accuracy as a function of encoder feed-forward block layer dimension, $\lambda_{\mathrm{ff}}$, for 100 classes. Test set performance appears to increase with the number of neurons in the feed-forward block layer.

**Figure 7:**
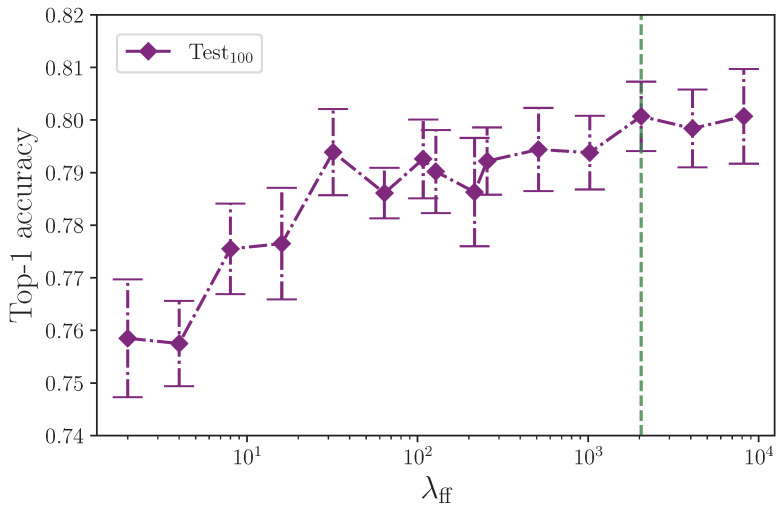
Log-linear plot showing mean top-1 accuracy as a function of encoder feed-forward block layer dimension, $\lambda_{\mathrm{ff}}$, for 100 classes. Only the test set results are shown, which more clearly shows that the test set performance increases with the number of neurons in the feed-forward block layer until it appears to plateau, over the range tested, at approximately 2048 neurons. This is marked with a green dashed line.

The effect that the number of neurons in the encoder feed-forward block layers has on model performance is surprising. Figure 6 shows the test set performance appears to rise in line with the feed-forward layer dimension. Figure 7 isolates the test set results and rescales them to give a clearer picture of what appears to be happening. We could expect an inflated layer dimension to increase overfitting on the training set, thereby impeding the ability of the model to generalise—especially so in the case of our model, which is clearly already overfitting—and conversely, we could reasonably expect a reduced layer dimension bottleneck to help mitigate overfitting, but this is not the outcome we observe with the experiments conducted. Model performance on the test set trends upwards with an increase in neurons up to the experimental limit of $\lambda_{\mathrm{ff}}=8192$, having plateaued at around $\lambda_{\mathrm{ff}}=2048$, with little performance difference in this range of dimensions. For convenience, this is marked in Figure 7 with a green dashed line. Both increasing the number of neurons beyond our limit of $\lambda_{\mathrm{ff}}=8192$ and the number of experiments per configuration would provide insight for determining the point at which the encoder feed-forward block layer dimension begins to impact on performance from overfitting the training dataset split to the detriment of generalisation to the test set. One possible explanation for the observed behaviour is that overfitting on the training set benefits some classes in the test set that have very similar equivalent training set examples. If this is the case, through increasingly memorising those training set class examples, the model more easily recognises very similar examples in the test set. It is worth noting that we do not see the same behaviour in the validation set, which could contain class examples with enough difference within each class when compared to the training set. This presents an opportunity for further analysis beyond the scope of this study, and would perhaps benefit from the expertise of someone proficient in ASL. What we find here is not dissimilar to the batch size experiment where an effect that we would expect to harm a more balanced dataset is not observed.

**Figure 8:**
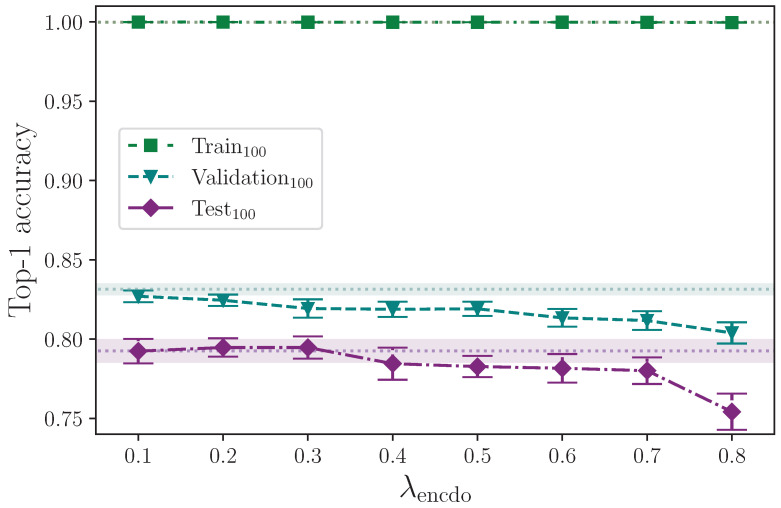
Plot showing mean top-1 accuracy as a function of encoder dropout probability, $\lambda_{\mathrm{encdo}}$, for 100 classes. Despite model performance on the test set appearing to peak at approximately $\lambda_{\mathrm{encdo}}=0.3$, the uncertainty prevents the determination of a clearly optimal value. Dropout probabilities $\lambda_{\mathrm{encdo}}>0.3$ appear to impede performance on the test set. The dashed lines with shaded areas indicate the respective mean top-1 accuracy and associated uncertainty for 16 experiments with no encoder dropout applied.

Figure 8 shows that applying dropout to the encoder appears to have little effect on model performance for $\lambda_{\mathrm{encdo}}\leq 0.3$. There is a slight improvement in test set performance for the range $0.2\leq \lambda_{\mathrm{encdo}}\leq 0.3$ compared with lower values of $\lambda_{\mathrm{encdo}}$, but given the experimental uncertainty of the measurements, no optimal value up to $\lambda_{\mathrm{encdo}}=0.3$ can be determined. Dropout probabilities greater than $\lambda_{\mathrm{encdo}}=0.3$, however, show a marked decline in performance, with the most severe reduction in accuracy at the upper limit $\lambda_{\mathrm{encdo}}=0.8$, which is unsurprising. Dropout also appears to reduce validation set performance across the spectrum. This is notable because dropout is generally considered advantageous to training deep neural networks [24,25]. One possible explanation is that whereas techniques like $\ell_{2}$ parameter regularisation encourage a spread of activations with no heavy dependencies on specific neural pathways, other techniques, like dropout, encourage neural pathway redundancy, which may still favour strong (but redundant) activation pathways, and because $\ell_{2}$ parameter regularisation is active by default throughout all dropout experiments, the two techniques could be in conflict when dropout is applied. Again, this presents another opportunity for a future study to measure the effect of applying a range of dropout probabilities against a range of $\ell_{2}$ parameter regularisation values, including none. It is, of course, also likely that the small dataset size plays an important role in the effect dropout has for improving the model’s capacity to generalise to unseen data.

**Figure 9:**
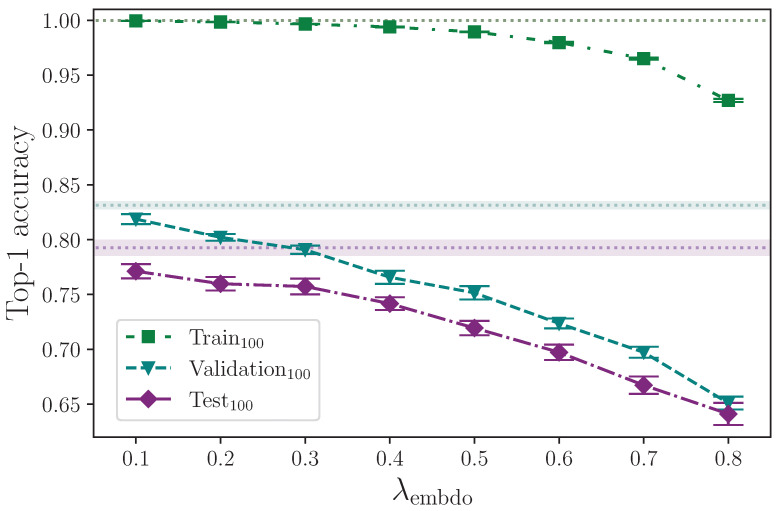
Plot showing mean top-1 accuracy as a function of embedding dropout probability, $\lambda_{\mathrm{embdo}}$, for 100 classes. It is clear that no amount of embedding dropout is beneficial for model performance on any of the dataset splits. The dashed lines with shaded areas indicate the respective mean top-1 accuracy and associated uncertainty for 16 experiments with no embedding dropout applied.

Dropout is applied to the training dataset split input embeddings as an analogue to neuron dropout in the encoder, but as can be seen in Figure 9, it is clear that no amount of embedding dropout is beneficial for model performance on any of the dataset splits. This categorically rules out embedding dropout as a valid strategy to reduce overfitting.

**Figure 10:**
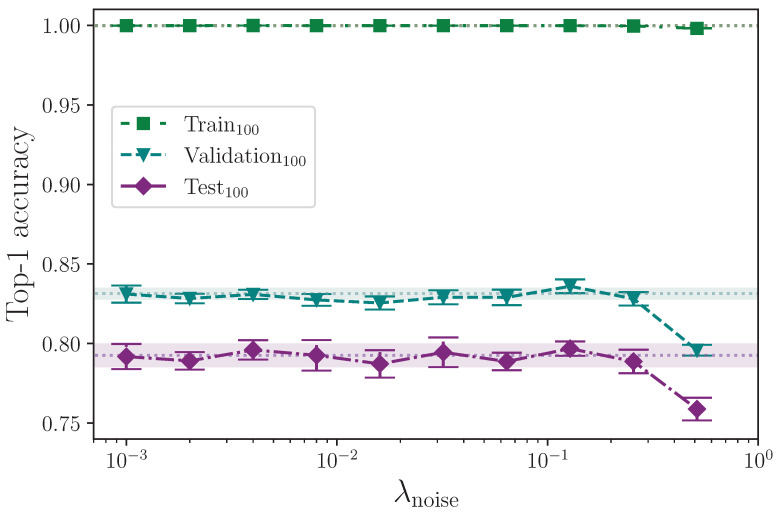
Log-linear plot showing mean top-1 accuracy as a function of noise augmentation, $\lambda_{\mathrm{noise}}$, for 100 classes. Given the experimental uncertainty, augmenting the data with noise appears to provide no measurable positive effect on model performance, with the effect being clearly negative beyond $\lambda_{\mathrm{noise}}=0.256$. The dashed lines with shaded areas indicate the respective mean top-1 accuracy and associated uncertainty for 16 experiments with no augmentation applied.

Augmenting the training dataset split by adding noise has no clear measurable effect on model performance for levels of noise $\lambda_{\mathrm{noise}}\leq 0.256$. The values of $\lambda_{\mathrm{noise}}$ quoted correspond with the maximum absolute value of injected noise per keypoint in a batch. Random batching means that, per epoch, batches will likely be included that contain little to no noise at all being applied. Figure 10 shows that once the level of injected noise goes above a maximum value of approximately $\lambda_{\mathrm{noise}}=0.256$, the amount of noisy data begins to overwhelm the less noisy data, which impedes the model’s ability to generalise to the test dataset split. The outcome of these experiments is in contrast to the study conducted by Bohacek and Hruz [40], who quote a measured increase in accuracy on 100 classes from 62.79% to 63.18% by augmenting with added noise.

**Figure 11:**
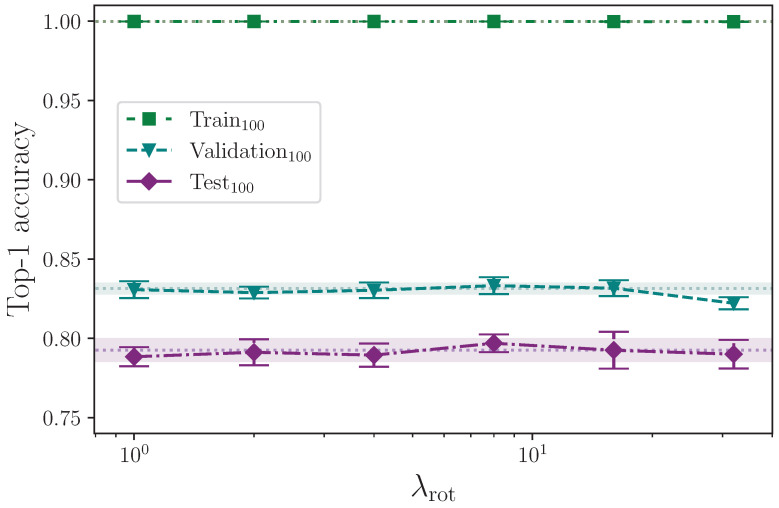
Log-linear plot showing mean top-1 accuracy as a function of rotation augmentation, $\lambda_{\mathrm{rot}}$, for 100 classes. Given the experimental uncertainty, it is not possible to claim a directly observable positive effect from augmenting with rotation of keypoints. The dashed lines with shaded areas indicate the respective mean top-1 accuracy and associated uncertainty for 16 experiments with no augmentation applied.

Augmenting the training dataset split by arbitrarily, but uniformly, rotating the keypoints about the origin, up to a maximum rotation value, appears to provide no real positive or negative effect on model test set performance, across the entire range of rotation values tested, $0\leq \lambda_{\mathrm{rot}}\leq 32$ (see Figure 11). Taking our best mean top-1 accuracy score from the rotation experiments, where $\lambda_{\mathrm{rot}}=8$, and again comparing with the study performed by Bohacek and Hruz [40], we see an increase of ∼0.43% from augmenting with rotation, whereas they report an increase in accuracy of 2.27%. It is possible that differences in implementation can cause some discrepancy, but with a measured experimental uncertainty of ∼0.56% relating to our best mean score, we conclude that we observe no measurable effect from augmenting with rotation.

**Figure 12:**
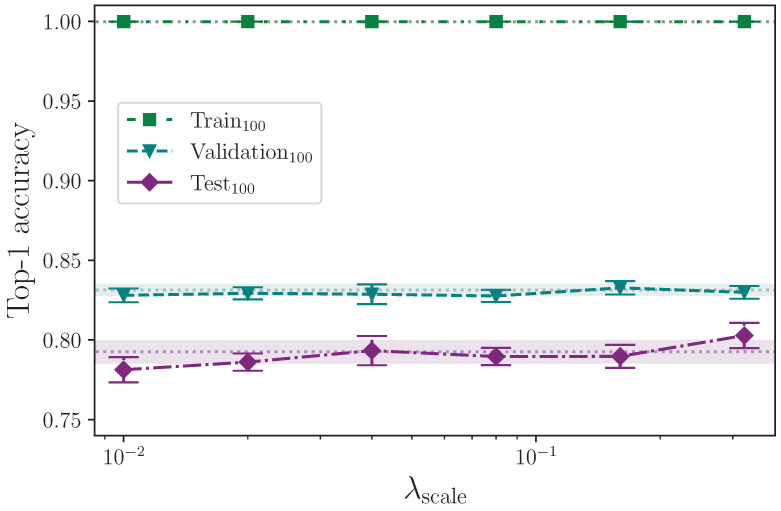
Log-linear plot showing mean top-1 accuracy as a function of scaling augmentation along the *x*-axis, $\lambda_{\mathrm{scale}}$, for 100 classes. Excluding the accuracy results for both extreme hyperparameter values, no clear measurable effect is observed. The measured uncertainties of those extremes, however, do fall within the measured uncertainty of the baseline experiments, indicated by the respective shaded areas, thereby preventing a clear positive or negative performance impact being observed. The dashed lines with shaded areas represent the respective mean top-1 accuracy and associated uncertainty for 16 experiments with no augmentation applied.

**Figure 13:**
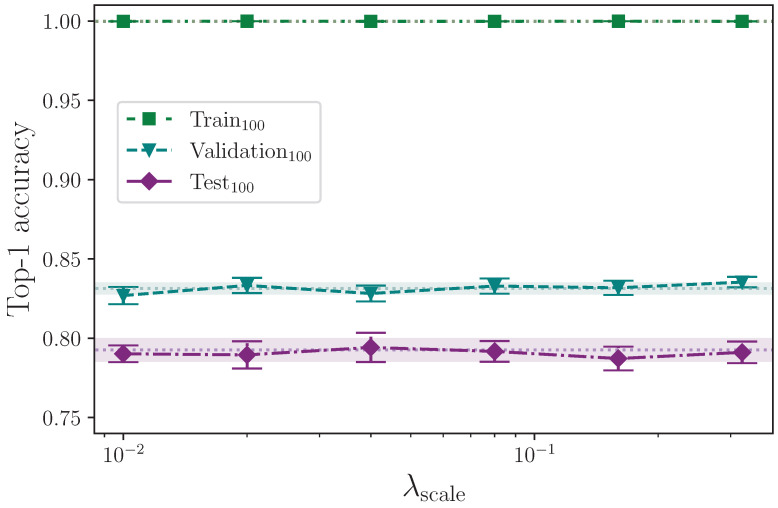
Log-linear plot showing mean top-1 accuracy as a function of scaling augmentation along the *y*-axis, $\lambda_{\mathrm{scale}}$, for 100 classes. There is no observable effect on test set performance from any scaling hyperparameter value $\lambda_{\mathrm{scale}}$. The dashed lines with shaded areas indicate the respective mean top-1 accuracy and associated uncertainty for 16 experiments with no augmentation applied.

**Figure 14:**
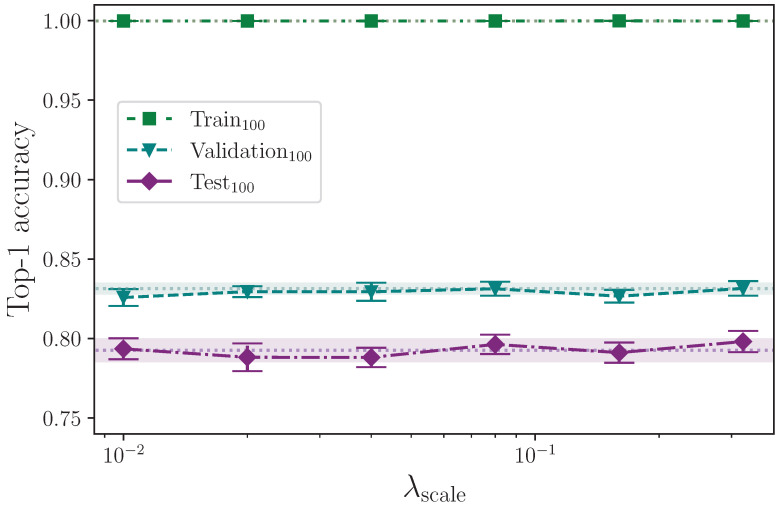
Log-linear plot showing mean top-1 accuracy as a function of scaling augmentation along the *x*- and *y*-axes, $\lambda_{\mathrm{scale}}$, for 100 classes. There is no observable effect on test set performance from any scaling hyperparameter value $\lambda_{\mathrm{scale}}$. The dashed lines with shaded areas indicate the respective mean top-1 accuracy and associated uncertainty for 16 experiments with no augmentation applied.

Analysing the three groups of scaling experiments together, we can see in Figure 12, Figure 13 and Figure 14 that augmenting the training dataset split by scaling separately along the *x*- and *y*-axes, and together on both axes, over the selected values, $0.00\leq \lambda_{\mathrm{scale}}\leq 0.32$, offers no test set performance enhancement. With the exception of the extremes of the tested values $\lambda_{\mathrm{scale}}=0.01$ and $\lambda_{\mathrm{scale}}=0.32$ along the *x*-axis, all results fall within the baseline uncertainty where no augmentation is applied. The uncertainties associated with the results from the experiments with $\lambda_{\mathrm{scale}}=0.01$ and $\lambda_{\mathrm{scale}}=0.32$ do, however, intersect, so no firm conclusion can be drawn. The rationale for this stems from the results for the superficially anomalous lowest value $\lambda_{\mathrm{scale}}=0.01$; augmenting by a maximum scaling value of $\lambda_{\mathrm{scale}}=0.01$ means the difference between keypoints that receive no *x*-axis perturbation and those that do is minuscule compared with those that, during random batching, receive a much larger range of perturbation values (e.g., $\lambda_{\mathrm{scale}}=0.08$) where the performance is somewhat increased compared with the baseline. The deviation from the mean at this lowest extreme is commensurate with the deviation at the upper extreme of $\lambda_{\mathrm{scale}}=0.32$, and we therefore conclude that the variation observed shows no real impact on model performance across the entire range of $\lambda_{\mathrm{scale}}$ in all axes other than perhaps reducing the stability of the model.

**Figure 15:**
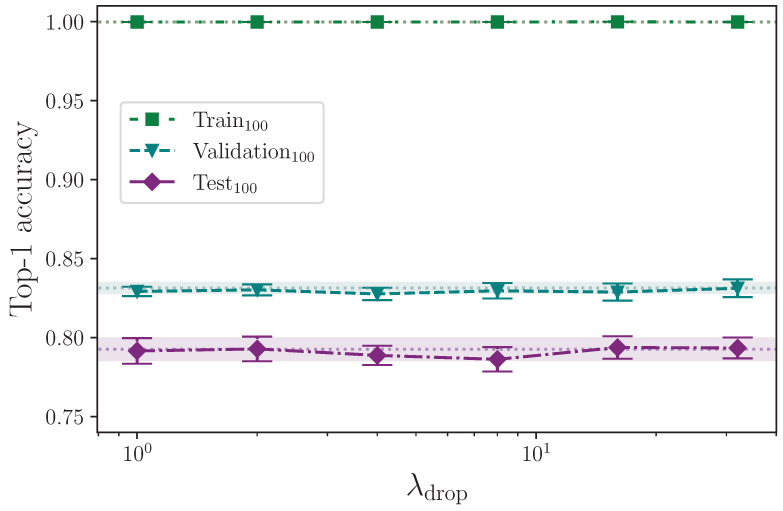
Log-linear plot showing mean top-1 accuracy as a function of the drop frames augmentation, $\lambda_{\mathrm{drop}}$, for 100 classes. Given the experimental uncertainty, it is not possible to claim a directly observable positive effect from augmenting by inserting an element of randomisation by dropping arbitrary frames from each sequence in a batch. The dashed lines with shaded areas indicate the respective mean top-1 accuracy and associated uncertainty for 16 experiments with no augmentation applied.

The subtle effect of simulating increasing the speed of movement in various places by dropping random frames, per batch, up to the configured maximum set by $\lambda_{\mathrm{drop}}$, appears to have no effect at all on model performance over the range of hyperparameter values tested. Figure 15 clearly shows no reduction in overfitting nor change in either validation or test set performance. We are somewhat surprised that this technique does not work as expected, and this is perhaps because the effect is too subtle. Increasing the range of values $\lambda_{\mathrm{drop}}$ can take may produce more positive results. Better still would be to intelligently remove frames using real analysis of the motion of various keypoints throughout a given sign, and thereby, for example, reducing the length of time a particular hold lasts, or speeding up the motion from rest to sign by differing amounts. But dramatically altering the sign sequences begins to encroach on synthetic sign dataset creation, and as previously stated, we recommend the involvement of (preferably Deaf) expert signers when manipulating signs beyond the basic techniques we have employed. We do not do so simply because we are not Deaf.

**Figure 16:**
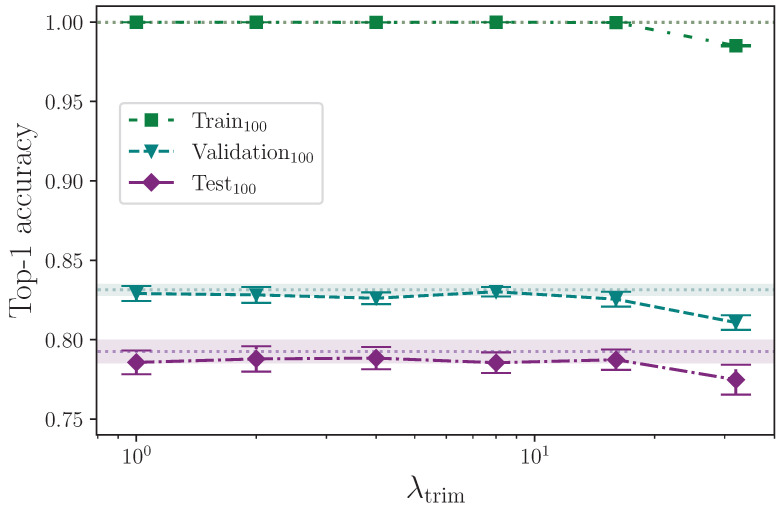
Log-linear plot showing mean top-1 accuracy as a function of the trim start augmentation, $\lambda_{\mathrm{trim}}$, for 100 classes. Given the experimental uncertainty, it is not possible to claim a directly observable positive effect from augmenting by inserting an element of randomisation by trimming arbitrary frames from the start of each sequence in a batch. The dashed lines with shaded areas indicate the respective mean top-1 accuracy and associated uncertainty for 16 experiments with no augmentation applied.

With the intention of both helping to remove some of the silence before a sign begins and to alter the time at which a sign does begin, such that more variety is introduced into the dataset, it is clear that the trim start augmentation becomes detrimental beyond a certain point, as Figure 16 clearly illustrates. Every mean experiment result sits towards the lower bound of the uncertainty of the baseline, up until $\lambda_{\mathrm{trim}}=0.16$, after which (at $\lambda_{\mathrm{trim}}=0.32$) performance clearly degrades across all dataset splits. This is almost certainly because a significant proportion of signs are being truncated with a sufficiently high $\lambda_{\mathrm{trim}}$. As with the drop frames experiment, performing this kind of augmentation more intelligently, with perhaps a mechanism to detect the start of the sign to ensure no essential frames are trimmed from the start of any given sequence, would likely yield better results. Likewise, extending the augmentation to detect the end of the sign would likely produce better results still [46].

**Figure 17:**
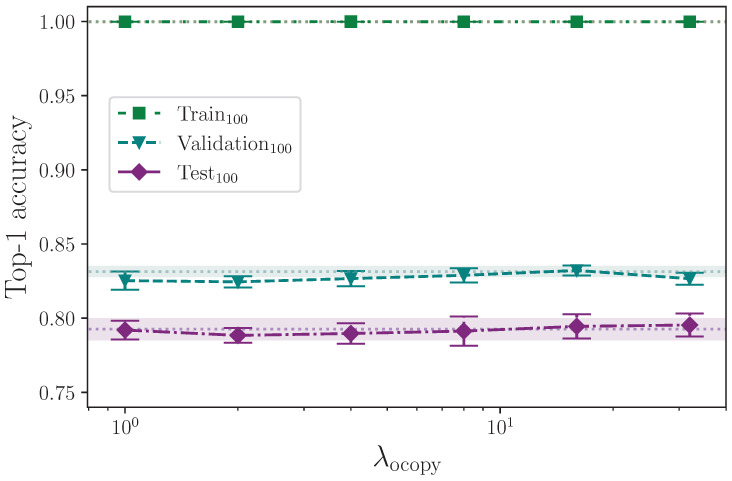
Log-linear plot showing mean top-1 accuracy as a function of the offset copy augmentation, $\lambda_{\mathrm{ocopy}}$, for 100 classes. Given the experimental uncertainty, it is not possible to claim a directly observable positive effect from augmenting by inserting copies of the first frame at the start of each sequence in a batch to delay the start of a sign. The dashed lines with shaded areas indicate the respective mean top-1 accuracy and associated uncertainty for 16 experiments with no augmentation applied.

**Figure 18:**
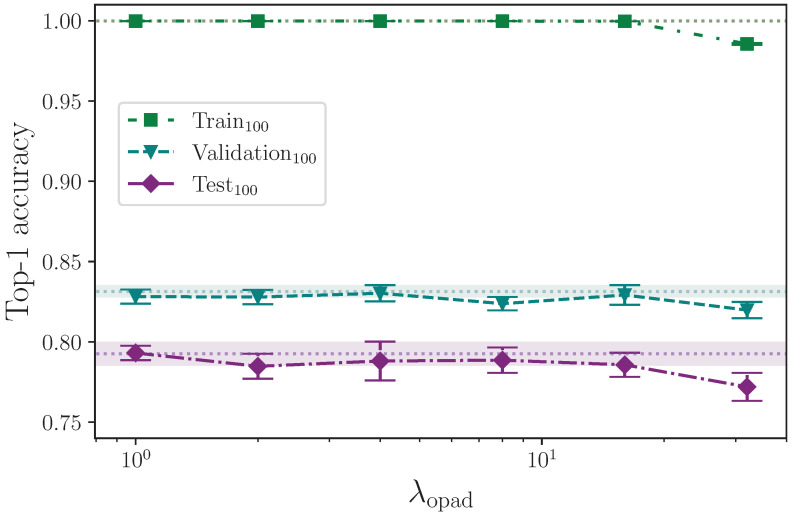
Log-linear plot showing mean top-1 accuracy as a function of the offset pad augmentation, $\lambda_{\mathrm{opad}}$, for 100 classes. Given the experimental uncertainty, it is not possible to claim a directly observable positive effect from augmenting by inserting blanks frames at the start of each sequence in a batch to delay the start of a sign, but only up until $\lambda_{\mathrm{opad}}=16$ after which it is clear that model performance is clearly degraded. The dashed lines with shaded areas indicate the respective mean top-1 accuracy and associated uncertainty for 16 experiments with no augmentation applied.

The offset augmentation techniques are very similar in that both delay the effective start of a given sign by inserting leading frames and reducing the length of the sequences as required to keep them constant. The only difference is that the offset copy augmentation inserts copies of the first frame data, whereas offset pad inserts empty frames. Referring to Figure 17 and Figure 18, it is clear that neither reduce overfitting to the benefit of the model’s ability to generalise to the validation and test sets, with the offset pad augmentation (which inserts blank frames) actively harming the model beyond $\lambda_{\mathrm{opad}}=16$. The conclusion that we are able to draw here is that some representative data, even if static, is better than no data at the beginning of a sign sequence. Given that all sequences are padded to a fixed length with zero values for the keypoint coordinates, and the sequence lengths—and by extension, sequence masks—are not used in the encoder, it would be interesting to pad sequences to the maximum sequence length with static copies of the final frame rather than empty values and measure the outcome.

We have seen from the experiments conducted so far that randomised seeds produce a range of results for experiments that are otherwise identical in configuration, and we show that it is necessary to repeat experiments many times to produce a mean value with associated measurement uncertainty so that the impact a given hyperparameter has on outcome can be evaluated. If a particular hyperparameter value is expected to produce a positive or negative change in outcome, we should expect the same polarity in the outcome by repeating the same experiment over a range of fixed seeds. The consequence of this not being true is that changing the value of a hyperparameter and repeating a single experiment is no guaranteed indicator of the efficacy of that hyperparameter change, with either a fixed or randomised seed. More simply, the requirement for multiple experiments with different seeds does not go away.

**Figure 19:**
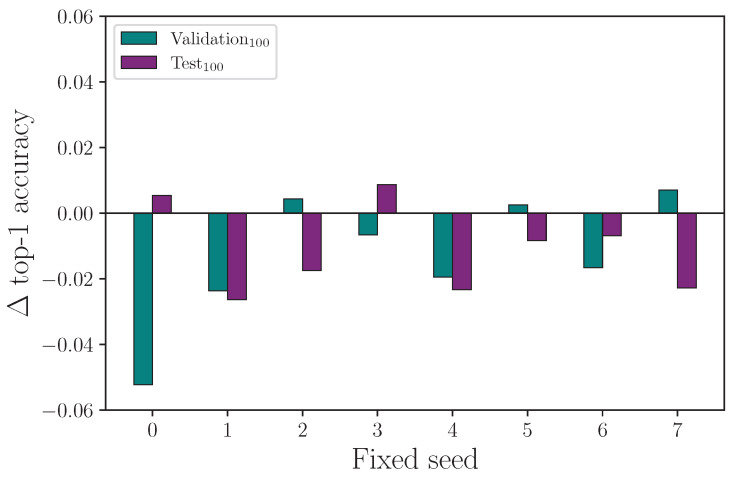
Impact of fixed seed on repeatability of singular feed-forward block layer dimension hyperparameter, $\lambda_{\mathrm{ff}}$, change. Each bar shows the difference between two experiments: the baseline hyperparameter value versus the altered hyperparameter value. A positive value indicates an improved accuracy score from the altered hyperparameter. The spread of values shows that the fixed seed can influence the impact of the hyperparameter value such that it is an insufficient way to test the outcome of hyperparameter changes with single experiments.

**Figure 20:**
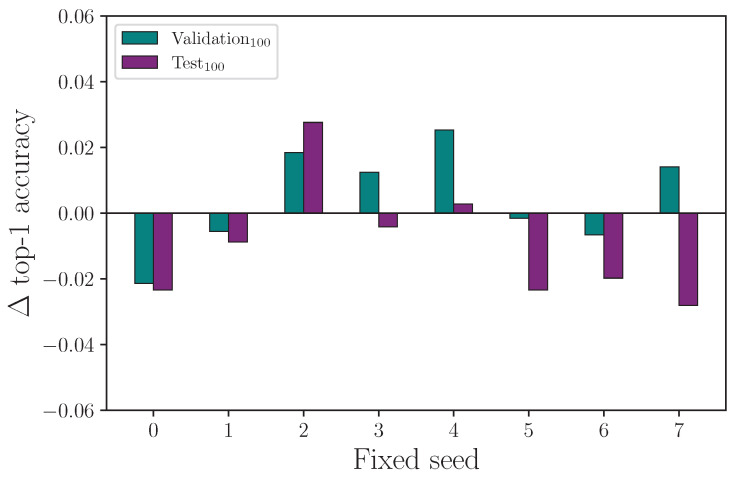
Impact of fixed seed on repeatability of singular rotation hyperparameter, $\lambda_{\mathrm{rot}}$, change. Each bar shows the difference between two experiments: the baseline hyperparameter value versus the altered hyperparameter value. A positive value indicates an improved accuracy score from the altered hyperparameter. The spread of values shows that the fixed seed can influence the impact of the hyperparameter value such that it is an insufficient way to test the outcome of hyperparameter changes with single experiments.

**Figure 21:**
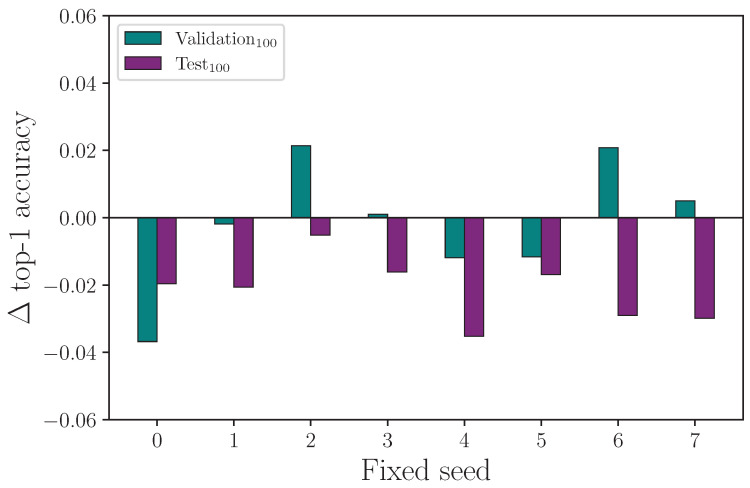
Impact of fixed seed on repeatability of singular encoder dropout hyperparameter, $\lambda_{\mathrm{encdo}}$, change. Each bar shows the difference between two experiments: the baseline hyperparameter value versus the altered hyperparameter value. A positive value indicates an improved accuracy score from the altered hyperparameter. The spread of values shows that the fixed seed can influence the impact of the hyperparameter value such that it is an insufficient way to test the outcome of hyperparameter changes with single experiments.

Reviewing the results from the fixed-seed comparison on singular hyperparameters experiment, we find evidence that fixing seeds does not lead to single-experiment results that show a clear outcome from a single hyperparameter change. For the experiment that compares training runs using the same fixed seeds for $\lambda_{\mathrm{ff}}=108$ (the control) and $\lambda_{\mathrm{ff}}=2048$, we plot the change in outcome for the validation and test sets for each given fixed seed such that a positive value means an improved accuracy was observed. We omit the training set outcomes because the difference is negligible. Figure 19 shows clearly that both the validation and test set accuracy scores can change considerably—and change polarity—when the exact same experiment is repeated with a different fixed seed. Even more notable here is that while Table 7 shows the mean top-1 accuracy after 16 experiments has $\lambda_{\mathrm{ff}}=2048$ performing better than $\lambda_{\mathrm{ff}}=108$ (the control), a naive interpretation of the results from this fixed-seed experiment would lead to the conclusion that an overall drop in performance is observed for $\lambda_{\mathrm{ff}}=2048$. Repeating the process for the experiment that compares training runs using the same fixed seeds for $\lambda_{\mathrm{rot}}=0$ (the control) and $\lambda_{\mathrm{rot}}=20$, we see a similar result (see Figure 20), with a spread of difference in outcome for both the validation and test set accuracy. Figure 21 shows the difference in outcome for the experiment with $\lambda_{\mathrm{encdo}}=0.0$ (the control) and $\lambda_{\mathrm{encdo}}=0.3$, but shows test set accuracy is reduced for each of the tested fixed seeds. Again, Table 8 shows the mean top-1 accuracy for $\lambda_{\mathrm{encdo}}=0.3$ after 16 experiments (each with a randomised seed) is marginally higher than $\lambda_{\mathrm{encdo}}=0.0$ on the same block of experiments. Running single experiments with any of the fixed seeds in our tested range, in this case, would lead to the erroneous conclusion that choosing $\lambda_{\mathrm{encdo}}=0.3$ would produce a worse test set accuracy than $\lambda_{\mathrm{encdo}}=0.0$, which we do not observe after 16 repeated experiments (see Table 8).

We now analyse the results from the fixed-seed comparison on normalisation experiment (see Table 18). Taking each group where the keypoints are normalised before augmentation versus renormalised after augmentation, we can perform an independent two-sample *t*-test and measure the statistical significance of the two distributions being comparable, i.e., no measurable difference was observed between the two groups. Insufficient resources prevent us from increasing the sample size, but nevertheless, we find the rotation experiment with $\lambda_{\mathrm{rot}}=20$ shows no significant difference between the two groups across the training, validation, and test set accuracies, with $p=2.377\times 10^{-1}$, $p=7.138\times 10^{-1}$, and $p=9.173\times 10^{-1}$, respectively. We find the same is true for the *x-y* scaling experiment with $\lambda_{\mathrm{scale}}=0.08$. With $p=7.266\times 10^{-1}$, $p=5.854\times 10^{-1}$, and $p=8.266\times 10^{-1}$, for the training, validation, and test splits, there is no significant difference found. We conclude that for the experiments conducted on our models, it is unlikely that renormalising after augmentation offers any performance gain. This is useful because it means the extra computational overhead of renormalising every batch can be avoided in future experiments that use these same techniques.

**Figure 22:**
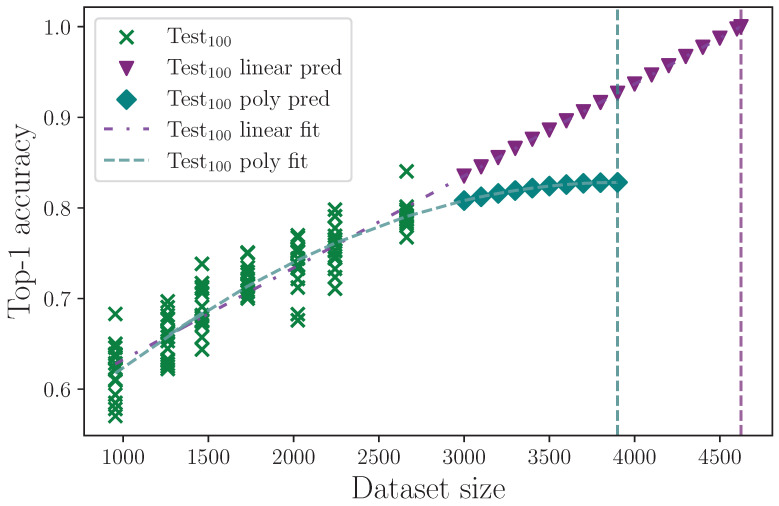
Top-1 test set accuracy as a function of dataset size, with conservative best- and worst-case fitted curves to estimate the effect increased dataset size has on accuracy. Predictions for the linear and polynomial of degree 2 fits have also been plotted. The hypothetical point, as predicted by the linear fit, at which the dataset size would allow for 100% accuracy is marked with a vertical purple dashed line, and the equivalent maximum accuracy point for the worst-case polynomial fit is marked with a vertical teal dashed line.

The dataset used in this study is small by deep learning standards, at only 2663 examples in the 100 sign classes group. Table 19 shows the impact that dataset size has on model performance by reducing the number of class examples by a ratio defined by the hyperparameter $\lambda_{\mathrm{red}}$, where $\lambda_{\mathrm{red}}=0.0$ means no reduction in size and $\lambda_{\mathrm{red}}=0.5$ would mean the number of class examples in each dataset split is halved. Reducing the dataset size shows a drop in top-1 test set accuracy proportional to the size, which is to be expected. To estimate the improvement that an increased dataset size could have on model performance, we can extrapolate to larger dataset sizes using regression analysis. As a conservative best-case estimate, we fit a linear trend to the full set of dataset reduction results and obtain the equation

$$y_{\mathrm{linear}}=1.015\times 10^{-4}x+5.309\times 10^{-1},$$

with coefficient of determination $R^{2}=8.402\times 10^{-1}$. For a worst-case estimate, we assume an increase in dataset size eventually asymptotes to a maximum accuracy value, at which the model is essentially saturated, and performance ceases to improve with increased dataset size. We therefore fit a polynomial of degree 2 and obtain the equation

$$y_{\mathrm{poly}}=-2.407\times 10^{-8}x^{2}+1.884\times 10^{-4}x+4.597\times 10^{-1},$$

with coefficient of determination $R^{2}=8.529\times 10^{-1}$. Using these equations, we predict mean accuracy at increased dataset sizes up until the model reaches 100% accuracy on the linear fit, and until the model reaches a peak on the polynomial fit. These results are shown in Figure 22, where the linear limit is found to be at a dataset size of 4624 example sequences, and for the polynomial fit, the peak is reached at 3900 example sequences. The linear and polynomial limits are marked with vertical dashed lines. We find the worst-case top-1 mean accuracy is approximately 83% given a sufficiently large dataset, which is a mean value improvement of approximately 3% on our best-performing model configuration. We therefore cautiously estimate that the dataset size would need to be doubled for this model to produce the best possible top-1 test set accuracy. Despite the analysis performed, we must also consider the change in balance that simply adding more example sequences would bring. If the dataset imbalance could be improved, so could our estimates and no doubt also our model performance. Notwithstanding these caveats, it is clearly demonstrated that the dataset size is the predominant limiting factor, given the correctness of our assumptions.

Finally, we note an improved maximum test set accuracy score for 100 classes of 0.8406, beating the previous accuracy score of 0.8316 [14]. This result was observed in the fixed-seed control experiment group with $\lambda_{\mathrm{seed}}=7$ (see Table 17), which is notable because, other than using a fixed-seed value, it utilises the common baseline hyperparameter configuration used throughout (see Table 2). We have reproduced and updated the results table from Woods and Rana [14] to include the best maximum top-1, top-5, and top-10 results for 100 classes from this ablation study (see Table 20). We have also included the best mean top-1 accuracy score, taking the associated top-5 and top-10 accuracy scores from that same experiment group. The best mean top-1 accuracy score was observed in the feed-forward block layer dimension experiment group with $\lambda_{\mathrm{ff}}=2048$.

For some of the augmentation techniques that show no impact on test set performance, it would have been better to repeat these experiments but by increasing the hyperparameter values until an effect is observed, positive or negative, or until the hyperparameter is exhausted (i.e., $\lambda_{\mathrm{rot}}$). It is possible that some of the augmentation techniques may still work, e.g., drop frames or offset copy, but only with sufficiently high associated hyperparameter values.

With many variations in the same signs, we conjecture that the difference between some of these same sign examples is greater than any basic augmentation technique allows to bridge the gap. For example, the WLASL-alt examples for the ASL sign show exhibit some variation that includes hand positions that are slightly to the left or right or to the front or at any intermediate angle. We hypothesise that the model would have to learn separate representations for distinct forms of the same signs, but proving this is beyond the scope of this study. The obvious conclusion is that—in accordance with our observations—minor perturbations like adding noise or rotating keypoints are insufficient compared with including more representative example sequences in the dataset that cover all sign variations, which would ultimately improve model performance. This is an important factor that differentiates image-based deep learning models [42,53] from sparse human pose estimation keypoint data-based models [14,40,41,42].

Performance gains made by optimising individual hyperparameters, which otherwise fall within the measured uncertainty across a sample of experiments, may have a stacking effect when combined with other optimised hyperparameters. Discovering these optimal combinations, however, would require a more thorough and exhaustive combinatorial search, which, given the exponentially explosive nature of all possible combinations, is impractical. This is where random hyperparameter search becomes the preferred choice [10].

## 5. Conclusions

In this study, we expand on the original sign language modelling study by Woods and Rana [14], by conducting a comprehensive, large-scale ablation study. We apply several regularisation and data augmentation techniques and find that none of these techniques dramatically reduces overfitting on the training set to the benefit of generalising to the test set. Our findings also appear to contradict previous similar studies that report performance gains from e.g., augmenting with noise. We find the boundaries of some hyperparameter values, and also that $\ell_{2}$ parameter regularisation via weight decay in the Adam optimiser offers the most significant benefit in improving model performance, but the gains are modest. We show that renormalising after augmentation is an unnecessary step that adds otherwise avoidable computational overhead. In addition, we further demonstrate the importance of conducting many experiments per configuration to enable reliable model performance evaluation, including showing that fixing random number generator seeds and repeating experiments once after a hyperparameter change is inadequate for determining the impact of that new hyperparameter value. While we cannot speculate on how our findings affect other supervised learning models and datasets—which are largely determined empirically—this study can help inform future similar studies aiming towards optimal hyperparameter selection by providing an extensive list of available hyperparameters to test as well as an indication of the anticipated outcome for various hyperparameter values, especially for sparse data like variable-length sequences of human pose estimation keypoints. Our experiments with padding using copies of the first frame versus empty frames suggest some data are better than none when padding, even if the keypoints represented by the data are static, and with this in mind, we recommend this idea is extended to the general process of padding sequences and tested. We also recommend testing random sampling of sequence frames to determine if the benefits reported elsewhere in the literature can be realised with this model architecture. We provide strong evidence that the small dataset size is the predominant limiting factor, and we believe our methods would have a greater impact with an increased dataset size. Finally, we report an improved maximum top-1 accuracy score on 100 classes of 84% using the same configuration as the original study.

## Figures and Tables

**Table 1:** Experiment dataset splits showing number of examples per split for 100 classes.

Training Examples	Validation Examples	Testing Examples	Total Examples
1842	418	403	2663

**Table 2:** Common baseline experiment parameters.

Parameter	Value (s)
Class count	100
Seed initialisation	Random
Epoch count	200
Renormalise after augmentation	False
Weight initialisation	Xavier uniform
Xavier uniform gain	1.0
Encoder $d_{\mathrm{model}}$	108
Encoder layers	1
Encoder attention heads	4
Encoder $d_{\mathrm{ff}}$	108
Encoder dropout	0.0
Encoder activation function	ReLU
Encoder norm first	False
Embedding dropout	0.0
Train/validation/test batch size	64
Batching method	Random batching
Reduced dataset ratio	0.0
Centroid keypoint index	1
Norm. scale *x* keypoint indices	{2, 5}
Norm. scale *y* keypoint indices	{0, 1}
Selected keypoints	All 54
$\ell_{1}$ parameter regularisation	False
$\ell_{2}$ parameter regularisation	True (via Adam weight decay)
Augment data	False
Optimiser	Adam
Adam weight decay	0.001
Adam $\beta_{1}$	0.9
Adam $\beta_{2}$	0.98
Adam ϵ	0.000000001
Learning rate	0.0001
Learning rate scheduler	Cosine annealing w/warm restarts
Cosine annealing $T_{0}$	10
Loss function	Cross-entropy loss

**Table 3:** Random batching vs. single-pass experiment mean top-1 accuracy for 100 classes. Highest values are shown in bold.

Batching Method	Epochs	Train Top-1 Accuracy	Validation Top-1 Accuracy	Test Top-1 Accuracy
Random	200	0.9998±0.0000	0.8314±0.0038	0.7926±0.0075
Single pass	200	0.8670±0.0032	0.6939±0.0072	0.6901±0.0078
Single pass	500	0.9993±0.0001	0.7859±0.0064	0.7646±0.0060
Single pass	1000	0.9995±0.0000	0.8098±0.0066	0.7961±0.0047
Single pass	1500	0.9995±0.0000	0.8165±0.0031	0.7940±0.0064

**Table 4:** Batch size experiment mean top-1 accuracy for 100 classes. Highest values are shown in bold.

Batch Size $\lambda_{\mathrm{batch}}$	Train Top-1 Accuracy	Validation Top-1 Accuracy	Test Top-1 Accuracy
16	0.9997±0.0001	0.8294±0.0050	0.7879±0.0075
32	0.9998±0.0000	0.8304±0.0055	0.7914±0.0071
64	0.9998±0.0000	0.8314±0.0038	0.7926±0.0075
128	0.9999±0.0000	0.8305±0.0031	0.7859±0.0073
256	0.9999±0.0000	0.8154±0.0041	0.7864±0.0074
512	0.9998±0.0000	0.8005±0.0063	0.7806±0.0081
1024	0.9986±0.0002	0.7904±0.0068	0.7799±0.0072
2048	0.3205±0.0055	0.2481±0.0091	0.2770±0.0047

**Table 5:** Learning rate experiment mean top-1 accuracy for 100 classes. Highest values are shown in bold.

Learning Rate $\lambda_{\mathrm{lr}}$	Train Top-1 Accuracy	Validation Top-1 Accuracy	Test Top-1 Accuracy
0.00001	0.9592±0.0032	0.7516±0.0074	0.7386±0.0082
0.00005	0.9999±0.0000	0.8200±0.0054	0.7875±0.0081
0.00010	0.9998±0.0000	0.8314±0.0038	0.7926±0.0075
0.00050	0.9995±0.0001	0.8279±0.0046	0.7952±0.0081
0.00100	0.9991±0.0001	0.8184±0.0043	0.7866±0.0064
0.00500	0.9826±0.0005	0.7458±0.0072	0.7062±0.0109
0.01000	0.9405±0.0011	0.6995±0.0054	0.6487±0.0086
0.05000	0.4365±0.0240	0.4034±0.0174	0.3607±0.0198

**Table 6:** $\ell_{1}$ and $\ell_{2}$ parameter regularisation experiment mean top-1 accuracy for 100 classes. Entries marked with – indicate no corresponding parameter regularisation is applied. Highest values are shown in bold.

$\ell_{1}\mathrm{Norm}$ $\lambda_{\ell_{1}}$	$\ell_{2}\mathrm{Norm}$ $\lambda_{\ell_{2}}$	Train Top-1 Accuracy	Validation Top-1 Accuracy	Test Top-1 Accuracy
–	–	0.9998±0.0000	0.8108±0.0044	0.7716±0.0113
0.00001	0.00100	0.9998±0.0000	0.8245±0.0049	0.7887±0.0075
0.00005	0.00100	0.9998±0.0000	0.8304±0.0065	0.7847±0.0082
0.00010	0.00100	0.9998±0.0000	0.8244±0.0038	0.7860±0.0094
0.00050	0.00100	0.9986±0.0002	0.8077±0.0054	0.7718±0.0080
0.00100	0.00100	0.9941±0.0005	0.7926±0.0046	0.7488±0.0091
0.00500	0.00100	0.9153±0.0026	0.7236±0.0053	0.6912±0.0075
0.01000	0.00100	0.5836±0.0293	0.4790±0.0222	0.4641±0.0236
0.05000	0.00100	0.0277±0.0013	0.0309±0.0024	0.0264±0.0038
0.10000	0.00100	0.0240±0.0011	0.0286±0.0020	0.0238±0.0039
–	0.00001	0.9998±0.0000	0.8197±0.0055	0.7732±0.0093
–	0.00005	0.9998±0.0000	0.8237±0.0063	0.7835±0.0131
–	0.00010	0.9998±0.0000	0.8295±0.0034	0.7887±0.0072
–	0.00050	0.9998±0.0000	0.8273±0.0051	0.7885±0.0065
–	0.00100	0.9998±0.0000	0.8314±0.0038	0.7926±0.0075
–	0.00500	0.9994±0.0001	0.8210±0.0044	0.7837±0.0084
–	0.01000	0.9946±0.0005	0.8125±0.0034	0.7742±0.0047
–	0.05000	0.8568±0.0107	0.7074±0.0083	0.6835±0.0095
–	0.10000	0.4705±0.0416	0.4063±0.0402	0.3726±0.0593

**Table 7:** Encoder feed-forward block layer dimension experiment mean top-1 accuracy for 100 classes. Highest values are shown in bold.

Encoder Feed-Forward Layer Dim $\lambda_{\mathrm{ff}}$	Train Top-1 Accuracy	Validation Top-1 Accuracy	Test Top-1 Accuracy
2	0.9998±0.0000	0.8096±0.0058	0.7585±0.0112
4	0.9998±0.0000	0.8118±0.0056	0.7575±0.0081
8	0.9998±0.0000	0.8185±0.0037	0.7755±0.0086
16	0.9998±0.0000	0.8176±0.0039	0.7765±0.0106
32	0.9998±0.0000	0.8181±0.0046	0.7939±0.0082
64	0.9999±0.0000	0.8265±0.0041	0.7861±0.0048
108	0.9998±0.0000	0.8314±0.0038	0.7926±0.0075
128	0.9999±0.0000	0.8291±0.0045	0.7902±0.0079
216	0.9998±0.0000	0.8274±0.0047	0.7863±0.0103
256	0.9998±0.0000	0.8270±0.0037	0.7922±0.0064
512	0.9998±0.0000	0.8231±0.0041	0.7944±0.0079
1024	0.9998±0.0000	0.8207±0.0038	0.7938±0.0070
2048	0.9997±0.0000	0.8119±0.0042	0.8007±0.0066
4096	0.9997±0.0000	0.8119±0.0051	0.7984±0.0074
8192	0.9997±0.0000	0.8122±0.0033	0.8007±0.0090

**Table 8:** Encoder dropout experiment mean top-1 accuracy for 100 classes. Highest values are shown in bold.

Encoder Dropout Probability $\lambda_{\mathrm{encdo}}$	Train Top-1 Accuracy	Validation Top-1 Accuracy	Test Top-1 Accuracy
0.0	0.9998±0.0000	0.8314±0.0038	0.7926±0.0075
0.1	0.9999±0.0000	0.8270±0.0037	0.7924±0.0077
0.2	0.9999±0.0000	0.8245±0.0036	0.7947±0.0058
0.3	0.9998±0.0000	0.8193±0.0058	0.7947±0.0070
0.4	0.9998±0.0000	0.8188±0.0048	0.7845±0.0101
0.5	0.9998±0.0000	0.8191±0.0045	0.7827±0.0067
0.6	0.9998±0.0000	0.8134±0.0056	0.7816±0.0090
0.7	0.9997±0.0000	0.8117±0.0059	0.7801±0.0084
0.8	0.9996±0.0001	0.8039±0.0067	0.7542±0.0114

**Table 9:** Embedding dropout experiment mean top-1 accuracy for 100 classes. Highest values are shown in bold.

Embedding Dropout Probability $\lambda_{\mathrm{embdo}}$	Train Top-1 Accuracy	Validation Top-1 Accuracy	Test Top-1 Accuracy
0.0	0.9998±0.0000	0.8314±0.0038	0.7926±0.0075
0.1	0.9994±0.0001	0.8187±0.0046	0.7712±0.0065
0.2	0.9984±0.0001	0.8021±0.0031	0.7598±0.0061
0.3	0.9966±0.0002	0.7908±0.0038	0.7573±0.0072
0.4	0.9939±0.0004	0.7657±0.0060	0.7417±0.0058
0.5	0.9894±0.0003	0.7515±0.0061	0.7194±0.0066
0.6	0.9797±0.0006	0.7236±0.0045	0.6973±0.0070
0.7	0.9650±0.0009	0.6973±0.0050	0.6673±0.0079
0.8	0.9269±0.0014	0.6510±0.0059	0.6411±0.0101

**Table 10:** Noise experiment mean top-1 accuracy for 100 classes. Highest values are shown in bold.

Maximum Noise $\lambda_{\mathrm{noise}}$	Train Top-1 Accuracy	Validation Top-1 Accuracy	Test Top-1 Accuracy
0.000	0.9998±0.0000	0.8314±0.0038	0.7926±0.0075
0.001	0.9998±0.0000	0.8311±0.0054	0.7918±0.0079
0.002	0.9998±0.0000	0.8283±0.0030	0.7891±0.0055
0.004	0.9999±0.0000	0.8309±0.0029	0.7960±0.0061
0.008	0.9998±0.0000	0.8274±0.0037	0.7926±0.0096
0.016	0.9998±0.0000	0.8255±0.0041	0.7872±0.0086
0.032	0.9998±0.0000	0.8291±0.0044	0.7945±0.0093
0.064	0.9998±0.0000	0.8290±0.0049	0.7887±0.0055
0.128	0.9998±0.0000	0.8360±0.0043	0.7968±0.0045
0.256	0.9996±0.0000	0.8281±0.0042	0.7887±0.0074
0.512	0.9981±0.0001	0.7958±0.0034	0.7588±0.0071

**Table 11:** Rotation experiment mean top-1 accuracy for 100 classes. Highest values are shown in bold.

Maximum Rotation $\lambda_{\mathrm{rot}}$	Train Top-1 Accuracy	Validation Top-1 Accuracy	Test Top-1 Accuracy
0	0.9998±0.0000	0.8314±0.0038	0.7926±0.0075
1	0.9998±0.0000	0.8307±0.0053	0.7884±0.0060
2	0.9998±0.0000	0.8288±0.0037	0.7912±0.0082
4	0.9998±0.0000	0.8303±0.0049	0.7894±0.0073
8	0.9998±0.0000	0.8332±0.0053	0.7969±0.0056
16	0.9997±0.0000	0.8316±0.0050	0.7925±0.0116
32	0.9996±0.0001	0.8221±0.0038	0.7900±0.0090

**Table 12:** Scale experiment mean top-1 accuracy for 100 classes along the *x*, *y*, and both axes. Highest values are shown in bold. The check mark indicates whether scaling was applied along the *x*- or *y*-axes.

Maximum Scaling $\lambda_{\mathrm{scale}}$	*x*-axis	*y*-axis	Train Top-1 Accuracy	Validation Top-1 Accuracy	Test Top-1 Accuracy
0.00			0.9998±0.0000	0.8314±0.0038	0.7926±0.0075
0.01	✓		0.9998±0.0000	0.8280±0.0043	0.7813±0.0079
0.02	✓		0.9998±0.0000	0.8293±0.0038	0.7861±0.0054
0.04	✓		0.9998±0.0000	0.8287±0.0062	0.7933±0.0092
0.08	✓		0.9998±0.0000	0.8276±0.0038	0.7896±0.0054
0.16	✓		0.9998±0.0000	0.8327±0.0042	0.7897±0.0072
0.32	✓		0.9998±0.0000	0.8299±0.0040	0.8028±0.0079
0.01		✓	0.9998±0.0000	0.8269±0.0054	0.7902±0.0053
0.02		✓	0.9999±0.0000	0.8333±0.0048	0.7895±0.0086
0.04		✓	0.9998±0.0000	0.8282±0.0050	0.7942±0.0092
0.08		✓	0.9998±0.0000	0.8329±0.0048	0.7917±0.0065
0.16		✓	0.9999±0.0000	0.8318±0.0045	0.7872±0.0075
0.32		✓	0.9998±0.0000	0.8354±0.0033	0.7911±0.0068
0.01	✓	✓	0.9998±0.0000	0.8258±0.0053	0.7935±0.0066
0.02	✓	✓	0.9998±0.0000	0.8295±0.0034	0.7882±0.0087
0.04	✓	✓	0.9998±0.0000	0.8294±0.0057	0.7881±0.0061
0.08	✓	✓	0.9998±0.0000	0.8313±0.0044	0.7963±0.0061
0.16	✓	✓	0.9999±0.0000	0.8266±0.0040	0.7911±0.0064
0.32	✓	✓	0.9998±0.0000	0.8315±0.0046	0.7981±0.0067

**Table 13:** Drop frames experiment mean top-1 accuracy for 100 classes. Highest values are shown in bold.

Maximum Frames to Drop $\lambda_{\mathrm{drop}}$	Train Top-1 Accuracy	Validation Top-1 Accuracy	Test Top-1 Accuracy
0	0.9998±0.0000	0.8314±0.0038	0.7926±0.0075
1	0.9998±0.0000	0.8292±0.0030	0.7915±0.0081
2	0.9998±0.0000	0.8302±0.0035	0.7928±0.0078
4	0.9998±0.0000	0.8276±0.0039	0.7887±0.0061
8	0.9998±0.0000	0.8296±0.0049	0.7862±0.0077
16	0.9999±0.0000	0.8288±0.0054	0.7937±0.0071
32	0.9998±0.0000	0.8312±0.0056	0.7934±0.0066

**Table 14:** Trim start experiment mean top-1 accuracy for 100 classes. Highest values are shown in bold.

Maximum Frames to Trim $\lambda_{\mathrm{trim}}$	Train Top-1 Accuracy	Validation Top-1 Accuracy	Test Top-1 Accuracy
0	0.9998±0.0000	0.8314±0.0038	0.7926±0.0075
1	0.9999±0.0000	0.8291±0.0047	0.7857±0.0075
2	0.9999±0.0000	0.8282±0.0050	0.7879±0.0080
4	0.9998±0.0000	0.8261±0.0037	0.7884±0.0070
8	0.9999±0.0000	0.8302±0.0030	0.7855±0.0065
16	0.9997±0.0000	0.8255±0.0047	0.7874±0.0064
32	0.9851±0.0005	0.8108±0.0046	0.7748±0.0094

**Table 15:** Offset frames copy experiment mean top-1 accuracy for 100 classes. Highest values are shown in bold.

Maximum Frames to Offset and Copy $\lambda_{\mathrm{ocopy}}$	Train Top-1 Accuracy	Validation Top-1 Accuracy	Test Top-1 Accuracy
0	0.9998±0.0000	0.8314±0.0038	0.7926±0.0075
1	0.9998±0.0000	0.8253±0.0061	0.7920±0.0063
2	0.9998±0.0000	0.8245±0.0038	0.7884±0.0050
4	0.9999±0.0000	0.8267±0.0051	0.7897±0.0069
8	0.9998±0.0000	0.8289±0.0048	0.7913±0.0098
16	0.9998±0.0000	0.8322±0.0034	0.7945±0.0082
32	0.9998±0.0000	0.8266±0.0041	0.7954±0.0078

**Table 16:** Offset frames pad experiment mean top-1 accuracy for 100 classes. Highest values are shown in bold.

Maximum Frames to Offset and Copy $\lambda_{\mathrm{opad}}$	Train Top-1 Accuracy	Validation Top-1 Accuracy	Test Top-1 Accuracy
0	0.9998±0.0000	0.8314±0.0038	0.7926±0.0075
1	0.9998±0.0000	0.8282±0.0044	0.7931±0.0045
2	0.9998±0.0000	0.8279±0.0044	0.7848±0.0078
4	0.9998±0.0000	0.8303±0.0051	0.7881±0.0121
8	0.9998±0.0000	0.8238±0.0042	0.7886±0.0079
16	0.9997±0.0000	0.8292±0.0061	0.7857±0.0075
32	0.9856±0.0006	0.8198±0.0051	0.7720±0.0087

**Table 17:** Fixed-seed comparison on singular hyperparameters experiment mean top-1 test accuracy for 100 classes. Highest values are shown in bold.

Seed	Control	$\lambda_{\mathrm{ff}}=2048$	$\lambda_{\mathrm{rot}}=20$	$\lambda_{\mathrm{scale}}=0.08$	$\lambda_{\mathrm{encdo}}=0.3$
0	0.7973	0.8027	0.7739	0.7876	0.7777
1	0.8170	0.7906	0.8082	0.8073	0.7964
2	0.7945	0.7770	0.8221	0.8105	0.7893
3	0.7813	0.7900	0.7771	0.8094	0.7652
4	0.7976	0.7743	0.8004	0.7918	0.7624
5	0.8188	0.8104	0.7954	0.7997	0.8019
6	0.8091	0.8022	0.7893	0.8084	0.7801
7	0.8406	0.8178	0.8125	0.8149	0.8107

**Table 18:** Fixed-seed comparison on normalisation experiment mean top-1 test accuracy for 100 classes. Highest values are shown in bold.

Seed	$\lambda_{\mathrm{rot}}=20$	$\lambda_{\mathrm{rot}}=20$ Renormalised	$\lambda_{\mathrm{scale}}=0.08$	$\lambda_{\mathrm{scale}}=0.08$ Renormalised
0	0.7739	0.7637	0.7876	0.7935
1	0.8082	0.8168	0.8073	0.7945
2	0.8221	0.8100	0.8105	0.8018
3	0.7771	0.7784	0.8094	0.8029
4	0.8004	0.7860	0.7918	0.7993
5	0.7954	0.7848	0.7997	0.8065
6	0.7893	0.8111	0.8084	0.8090
7	0.8125	0.8201	0.8149	0.8319

**Table 19:** Dataset size experiment mean top-1 test accuracy for 100 classes. Highest values are shown in bold.

Dataset Size Reduction Ratio $\lambda_{\mathrm{red}}$	Train Top-1 Accuracy	Validation Top-1 Accuracy	Test Top-1 Accuracy
0.0	0.9998±0.0000	0.8314±0.0038	0.7926±0.0075
0.1	1.0000±0.0000	0.7821±0.0052	0.7590±0.0118
0.2	1.0000±0.0000	0.7605±0.0058	0.7364±0.0132
0.3	1.0000±0.0000	0.7652±0.0060	0.7213±0.0079
0.4	1.0000±0.0000	0.7453±0.0102	0.6867±0.0119
0.5	1.0000±0.0000	0.7222±0.0097	0.6544±0.0123
0.6	1.0000±0.0000	0.7201±0.0095	0.6180±0.0145

**Table 20:** Updated table from Woods and Rana [14] showing best top-1, top-5, and top-10 test accuracy results for human pose-estimation-based sign language recognition using WLASL-based data. Highest values are shown in bold.

Model	10 Classes Top-*k*			50 Classes Top-*k*			100 Classes Top-*k*			300 Classes Top-*k*		
	**1**	**5**	**10**	**1**	**5**	**10**	**1**	**5**	**10**	**1**	**5**	**10**
Pose-TGCN [42]	–	–	–	–	–	–	0.5543	0.7868	0.8760	0.3832	0.6751	0.7964
Pose-GRU [42]	–	–	–	–	–	–	0.4651	0.7674	0.8566	0.3368	0.6437	0.7605
GCN-BERT [43]	–	–	–	–	–	–	0.6015	0.8398	0.8867	0.4216	0.7171	0.8093
SPOTER [40]	–	–	–	–	–	–	0.6318	–	–	0.4378	–	–
Sign2Pose [41]	–	–	–	–	–	–	0.8090	–	–	0.6421	–	–
Woods and Rana [14] (mean)	0.9395	0.9854	1.0000	0.8330	0.9634	0.9771	0.7993	0.9415	0.9619	0.6855	0.8920	0.9318
Woods and Rana [14] (max)	0.9688	1.0000	1.0000	0.8722	0.9746	0.9831	0.8316	0.9596	0.9733	0.7052	0.8999	0.9378
Ours (mean)	–	–	–	–	–	–	0.8007	0.9395	0.9600	–	–	–
Ours (max)	–	–	–	–	–	–	0.8406	0.9641	0.9795	–	–	–

## Data Availability

The WLASL dataset is available at https://dxli94.github.io/WLASL/ (accessed on 27 August 2023). The WLASL-alt dataset is available at https://dai.cs.rutgers.edu/dai/s/wlasl (accessed on 27 August 2023). The dataset splits used in this study are available at https://github.com/ltwoods/msl (accessed on 27 August 2023).

## Abbreviations

The following abbreviations are used in this manuscript:

ASL	American Sign Language
BERT	Bidirectional Encoder Representations from Transformers
GCN	graph convolution network
GRU	gated recurrent unit
NLP	natural language processing
ReLU	rectified linear unit
SPOTER	Sign POsebased TransformER
WLASL	Word-level American Sign Language dataset
WLASL-alt	Word-level American Sign Language alternative dataset

## Appendix A

**Table A1:** Random batching method top-1 accuracy results for 100 classes.

Train Top-1 Accuracy	Validation Top-1 Accuracy	Test Top-1 Accuracy
0.9999	0.8238	0.7964
0.9998	0.8366	0.7898
0.9998	0.8262	0.7804
0.9997	0.8376	0.7827
0.9998	0.8207	0.7877
0.9999	0.8274	0.7965
0.9999	0.8405	0.8404
0.9998	0.8417	0.7676
0.9999	0.8366	0.7844
0.9999	0.8347	0.7908
0.9999	0.8273	0.7984
0.9999	0.8347	0.7874
0.9998	0.8323	0.7983
0.9999	0.8376	0.7920
0.9997	0.8309	0.8014
0.9999	0.8132	0.7867

**Table A2:** Single pass batching method top-1 accuracy results for 100 classes.

Train Top-1 Accuracy	Validation Top-1 Accuracy	Test Top-1 Accuracy
0.9995	0.8134	0.7767
0.9995	0.8254	0.8139
0.9995	0.8206	0.7618
0.9995	0.8110	0.7866
0.9995	0.8230	0.8114
0.9995	0.8134	0.7916
0.9995	0.8134	0.7940
0.9995	0.8134	0.7940
0.9995	0.8278	0.7891
0.9995	0.8158	0.7916
0.9995	0.8158	0.7965
0.9995	0.8038	0.8015
0.9995	0.8182	0.7866
0.9995	0.8230	0.8065
0.9995	0.8086	0.7965
0.9995	0.8182	0.8065

**Table A3:** Parameter regularisation control top-1 accuracy results for 100 classes.

Train Top-1 Accuracy	Validation Top-1 Accuracy	Test Top-1 Accuracy
0.9999	0.7993	0.7197
0.9999	0.8051	0.7351
0.9998	0.7949	0.7520
0.9999	0.7972	0.7592
0.9998	0.8183	0.7953
0.9998	0.8092	0.7767
0.9997	0.8156	0.7797
0.9998	0.8223	0.7803
0.9999	0.8221	0.7893
0.9999	0.8158	0.7539
0.9998	0.8082	0.8021
0.9999	0.8118	0.7957
0.9999	0.8256	0.7817
0.9997	0.8109	0.7863
0.9998	0.8050	0.7589
0.9999	0.8110	0.7804

**Table A4:** Elastic net regularisation top-1 accuracy results for 100 classes.

Train Top-1 Accuracy	Validation Top-1 Accuracy	Test Top-1 Accuracy
0.9998	0.8024	0.8049
0.9999	0.8409	0.7863
0.9998	0.8170	0.7760
0.9998	0.8387	0.7871
0.9998	0.8215	0.7797
0.9997	0.8307	0.8086
0.9999	0.8322	0.7747
0.9999	0.8170	0.7808
0.9998	0.8305	0.8113
0.9998	0.8300	0.7844
0.9998	0.8231	0.7934
0.9999	0.8137	0.8070
0.9999	0.8253	0.7953
0.9998	0.8206	0.7545
0.9997	0.8163	0.7980
0.9998	0.8323	0.7770

**Table A5:** $\ell_{2}$ parameter regularisation top-1 accuracy results for 100 classes.

Train Top-1 Accuracy	Validation Top-1 Accuracy	Test Top-1 Accuracy
0.9999	0.8238	0.7964
0.9998	0.8366	0.7898
0.9998	0.8262	0.7804
0.9997	0.8376	0.7827
0.9998	0.8207	0.7877
0.9999	0.8274	0.7965
0.9999	0.8405	0.8404
0.9998	0.8417	0.7676
0.9999	0.8366	0.7844
0.9999	0.8347	0.7908
0.9999	0.8273	0.7984
0.9999	0.8347	0.7874
0.9998	0.8323	0.7983
0.9999	0.8376	0.7920
0.9997	0.8309	0.8014
0.9999	0.8132	0.7867
